# Investigating Causal Relationships Between Major Mental Disorders and Hypertension Subtypes and Their Complications: A Mendelian Randomization Study

**DOI:** 10.1111/gbb.70078

**Published:** 2026-11-08

**Authors:** Jingyang Su, Jialin Zhang, Yueyue Ding, Yongjun Ying

**Affiliations:** ^1^ Department of General Internal Medicine Tongde Hospital Affiliated to Zhejiang Chinese Medical University (Tongde Hospital of Zhejiang Province) Hangzhou China; ^2^ The Second Affiliated Hospital of Zhejiang Chinese Medical University (Xinhua Hospital of Zhejiang Province) Hangzhou China; ^3^ Department of Geriatrics Tongde Hospital Affiliated to Zhejiang Chinese Medical University (Tongde Hospital of Zhejiang Province) Hangzhou China

**Keywords:** bipolar disorder, hypertension, major depression, Mendelian randomization study, schizophrenia

## Abstract

The association between major psychiatric disorders (major depression, bipolar disorder, schizophrenia) and hypertension has long been of clinical and research interest. Using publicly available summary data from large‐scale genome‐wide association studies (GWASs), we selected, quality‐controlled, and clustered eligible genetic variants for the three psychiatric disorders and hypertension‐related outcomes. The inverse variance weighted (IVW) method was applied as the primary analysis, with a suite of sensitivity analyses including MR‐Egger regression, weighted median estimator (WME), and leave‐one‐out analysis performed to assess result robustness and detect potential horizontal pleiotropy. False discovery rate (FDR) correction was implemented to multiple testing. Bidirectional two‐sample Mendelian randomization (MR) identified a reciprocal causal association between major depression and essential hypertension. Genetically predicted major depression was significantly associated with an increased risk of essential hypertension [IVW: odds ratio (OR) 1.247, 95% confidence interval (CI) (1.117–1.393), *p* < 0.001; WME: OR 1.243, 95% CI (1.127–1.371), *p* < 0.001]. Conversely, genetic predisposition to essential hypertension also increased the risk of major depression (IVW: OR 1.028, 95% CI (1.007–1.050), *p* = 0.008), which remained significant after FDR correction. We found that bipolar disorder was causally associated with essential hypertension, hypertensive heart disease, and hypertensive renal disease (*p* < 0.05). In contrast, no causal associations were observed between schizophrenia and any hypertension subtypes or complications (*p* > 0.05). All sensitivity analyses confirmed the robustness of the primary effect estimates, with no evidence of significant horizontal pleiotropy or influential outliers. In conclusion, our study, conducted under the MR framework from the perspective of genetic susceptibility, supports a bidirectional causal relationship between major depression and essential hypertension.

## Graphical abstract

Based on the validity of the instrumental variable assumption, our Mendelian randomization analysis shows that the genetic predisposition to major depression is associated with an increased risk of essential hypertension and suggests a possible bidirectional effect between major depression and essential hypertension.

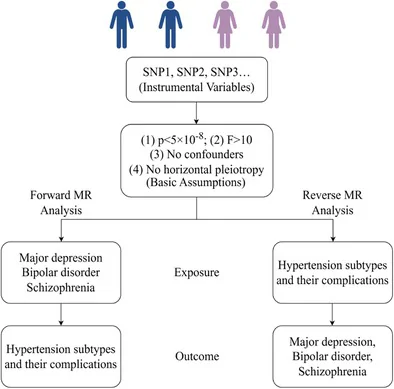

## 1 Introduction

Hypertension, its pre‐stage conditions, and distinct risk subtypes have emerged as a well‐recognized “silent killer” in global health. According to data from the World Health Organization (WHO) and its partner organizations, approximately 1.28 billion adults worldwide are living with hypertension [1], imposing substantial disease burden and economic strain on health systems and societies across the globe [2]. Further epidemiological data indicate that elevated blood pressure accounts for 7.7 to 10.4 million deaths globally on an annual basis [3]. Notably, around 8.5 million of these deaths are specifically attributed to hypertension‐related complications [1], including hypertensive heart disease, hypertensive encephalopathy, and hypertensive renal disease [4]. Alongside the rapidly rising burden of hypertension, the global prevalence of mental disorders has increased substantially from 654.8 million cases in 1990 to 970.1 million cases in 2019, representing a 48.1% relative growth over three decades [5]. Major depression, bipolar disorder, and schizophrenia are among the most prevalent and highly heritable mental disorders globally; these conditions contribute heavily to the global burden of disease, accounting for a total of 40.5% of disability‐adjusted life years attributed to all mental health conditions [5, 6]. Accumulating epidemiological evidence has established that these mental disorders not only correlate strongly with suicidal behavior but also significantly increase the risk of multiple cardiovascular diseases, including hypertension [6].

A growing body of evidence supports a potential causal association between mental disorders and hypertension, with multiple observational studies consistently reporting an elevated risk of incident hypertension among individuals with preexisting mental health symptoms, as well as a higher point prevalence of depression in patients with established hypertension [7, 8, 9, 10]. Existing evidence further suggests that comorbidity of mental disorders and hypertension substantially worsens long‐term patient mortality and health‐related quality of life, while shared genetic architecture and overlapping clinical phenotypes between depression, bipolar disorder, and other severe mental disorders imply the existence of common underlying pathogenic mechanisms [11, 12, 13, 14]. Collectively, current evidence points to a plausible bidirectional relationship, in which mental disorders may increase the subsequent risk of incident hypertension, and hypertension may, in turn, elevate the likelihood of new‐onset mental disorders.

After systematic literature review, we identified that two previously published Mendelian randomization (MR) studies reported a positive causal effect of depression on hypertension [15, 16]. However, these two MR studies found no statistically significant evidence that hypertension increases depression risk [15, 16], a finding that contradicts conclusions from earlier meta‐analyses [14]. We hypothesize that this apparent discrepancy may be partly explained by the failure of prior studies to distinguish between distinct hypertension subtypes (e.g., essential hypertension vs. secondary hypertension), which may have introduced non‐negligible outcome heterogeneity and biased the corresponding causal estimates. Hypertension is a clinically heterogeneous condition consisting of distinct phenotypic subtypes, including total hypertension, essential hypertension, secondary hypertension, and pregnancy hypertension, each with unique clinical presentations and distinct underlying pathophysiology mechanisms. Clarifying the causal associations between different psychiatric disorders and these specific hypertension subtypes, rather than treating hypertension as a single uniform disease entity, helps avoid ambiguous and inconsistent conclusions, and provides targeted evidence to support stratified clinical prevention and tailored management of these comorbid conditions. To date, systematic research investigating the potential causal relationships between the three most common mental disorders (major depression, bipolar disorder, and schizophrenia) and different hypertension subtypes and their complications remains scarce. This knowledge gap highlights the necessity of this study to clarify these relationships from a genetic perspective. Given the inherent limitations and practical challenges of conducting large‐scale clinical randomized controlled trials to address this question (including high costs and logistical complexity), we performed the current bidirectional two‐sample MR study to fill these unmet research gaps. Using publicly available summary‐level statistics from large‐scale genome‐wide association studies (GWASs), we systematically evaluated the bidirectional causal associations between three major mental disorders and multiple hypertension subtypes and their complications. MR is a robust randomized controlled trial (RCT) alternative that uses genetic variants as instrumental variables (IVs). By leveraging the randomness of germline genetic variation at fertilization and allele assortment during meiosis, MR methodology effectively minimizes confounding and reverse causality bias inherent in conventional observational studies [17]. Our findings will generate new, methodologically robust evidence to inform targeted prevention and optimized clinical management strategies for these highly prevalent comorbid conditions.

## 2 Methods

### 2.1 Study Design

Using publicly available data from large‐scale GWASs, we performed a bidirectional two‐sample MR analysis to investigate potential causal relationships between three common mental disorders (major depression, bipolar disorder, and schizophrenia) and hypertension subtypes and their complications (hypertension, essential hypertension, secondary hypertension, pregnancy hypertension, hypertensive heart disease, hypertensive renal disease, hypertensive encephalopathy). This analysis strictly adhered to the three core assumptions of MR (summarized in Figure 1), which form the methodological foundation for valid causal inference in the present study [18].

**FIGURE 1:**
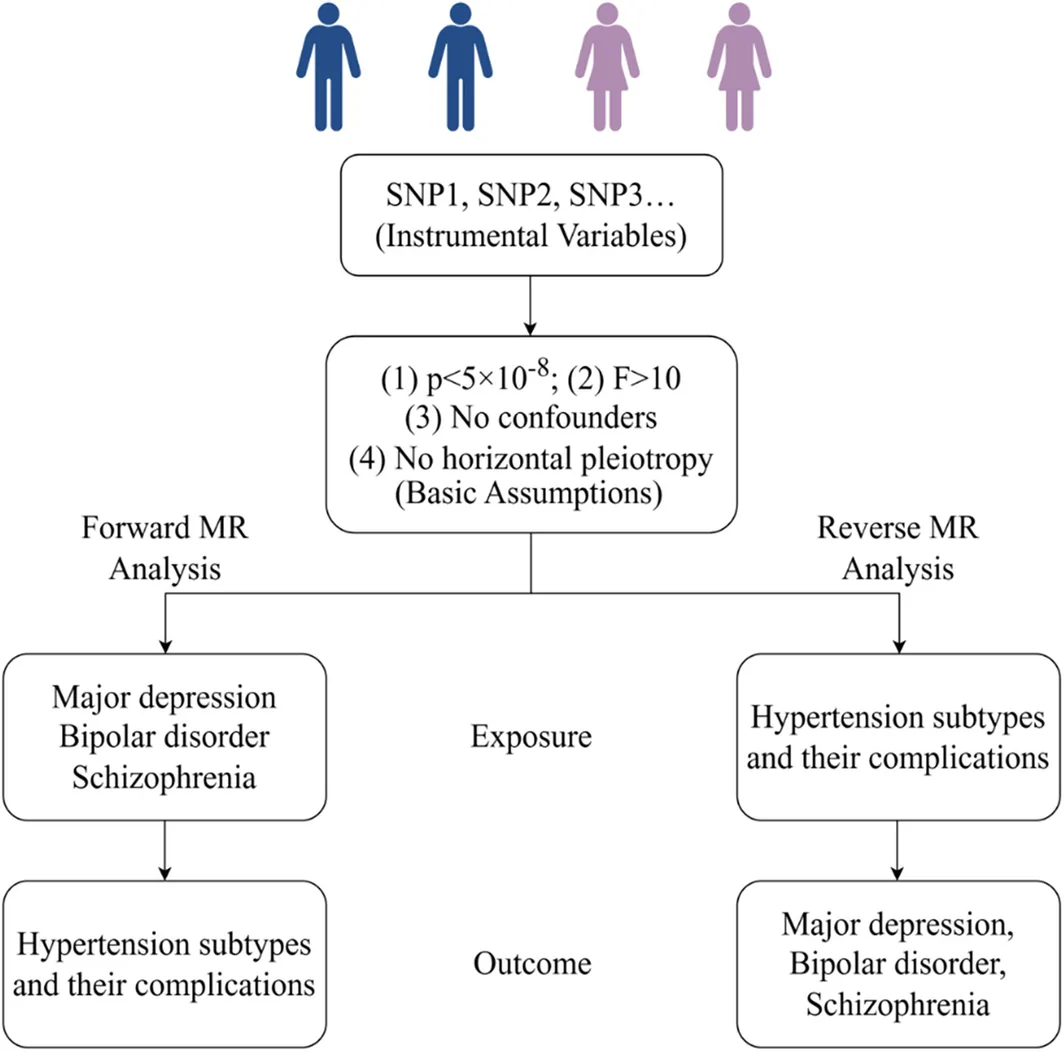
Flowchart of bidirectional Mendelian randomization.

This study follows the STROBE‐MR reporting guidelines to enhance transparency and reproducibility—key standards for high‐quality epidemiological research. All data utilized in the analysis are publicly available from established GWAS repositories, obviating the need for additional ethical approval or informed consent (as no primary data collection was involved).

### 2.2 Data Sources

Genetic data on mental disorders were retrieved from the Psychiatric Genomics Consortium (PGC) database—a leading repository for psychiatric genetic research. To eliminate confounding from population stratification, we restricted our analysis to study populations of exclusively European ancestry. Specifically, the major depression dataset [19] included 170,756 cases and 329,443 controls, while the bipolar disorder dataset [20] comprised 41,917 cases and 371,549 controls. And schizophrenia data were derived from Trubetskoy et al.'s study [21], which involved 76,755 cases and 243,649 controls.

GWAS data for various hypertension subtypes and their complications were acquired from the FinnGen database (https://r12.finngen.fi/), a population‐based resource for Finnish genetic research. A total of seven datasets were included to capture the heterogeneity of hypertension phenotypes. The genome‐wide association summary statistics for the overall hypertension phenotype were derived from a cohort of 500,264 participants. The remaining phenotypic datasets contained the following sample sizes: 132,515 cases and 345,634 controls for essential hypertension; 3388 cases and 225,810 controls for secondary hypertension; 20,405 cases and 261,659 controls for pregnancy‐induced hypertension; 11,728 cases and 345,634 controls for hypertensive heart disease; 1367 cases and 345,634 controls for hypertensive renal disease; and 124 cases and 453,828 controls for hypertensive encephalopathy. Detailed characteristics of all datasets (e.g., sample sizes, phenotypic definitions, datasets) are summarized in Table 1.

**TABLE 1:** Genetic data characteristics of mental disorders and hypertension subtypes/complications.

Trait	Source	Dataset	Case (*n*)	Control (*n*)	Sample size	Population
Major depression	PGC	ieu‐b‐102	170,756	329,443	500,199	European
Bipolar disorder	PGC	ieu‐b‐5110	41,917	371,549	413,466	European
Schizophrenia	PGC	ieu‐b‐5099	76,755	243,649	320,404	European
Hypertension	FinnGen	finngen_R12_I9_HYPTENS	154,630	345,634	500,264	European
Essential hypertension	FinnGen	finngen_R12_I9_HYPTENSESS	132,515	345,634	478,149	European
Secondary hypertension	FinnGen	finngen_R12_I9_HYPTENSEC	3388	252,810	256,198	European
Pregnancy hypertension	FinnGen	finngen_R12_O15_HYPTENSPREG	20,405	261,659	282,064	European
Hypertensive heart disease	FinnGen	finngen_R12_I9_HYPTENSHD	11,728	345,634	357,362	European
Hypertensive renal disease	FinnGen	finngen_R12_I9_HYPTENSRD	1367	345,634	347,001	European
Hypertensive encephalopathy	FinnGen	finngen_R12_I9_HYPERTENC	124	453,828	453,952	European

### 2.3 Selection of IVs

For forward MR analysis, mental disorders served as the exposure, with hypertension subtypes and their complications as outcomes. The reverse analysis swapped these roles (hypertension subtypes/complications as exposure, mental disorders as outcome). Single‐nucleotide polymorphism (SNPs) significantly associated with mental disorders (from the PGC database) or hypertension subtypes/complications (from the FinnGen database) were extracted using strict criteria to ensure SNP independence: *p* < 5 × 10^−8^, linkage disequilibrium [LD] *r* ^2^ < 0.001, genetic distance = 10,000 KB.

We implemented a rigorous multistep quality control workflow to ensure that the three core assumptions of the MR model were strictly met throughout the analysis process. First, we calculated the F‐statistic for each retained SNP using the formula F = *β* ^*2*^ */SE* ^*2*^ to verify the robust association between IVs and exposure traits. Only SNPs with an F‐statistic greater than 10 were included in subsequent analyses, as this widely accepted threshold effectively excludes potential weak instrument bias [22, 23]. This standard threshold reduces bias introduced by weak IVs, and the selected instrumental alleles follow the natural principle of random allelic assortment from parents to offspring, thereby substantially eliminating potential confounding effects introduced by post‐randomization environmental exposures. In addition, we adopted a standardized allele alignment procedure. We used the TwoSampleMR R package (version 0.6.18) to harmonize the directions of the selected SNP alleles between the exposure and outcome datasets, aligning the effect allele of each instrumental SNP consistently across the exposure and outcome GWAS summary statistics. This harmonization process unified the definitions of reference and alternate alleles across datasets, corrected strand ambiguity for palindromic SNPs by cross‐referencing corresponding allele frequency information, and excluded all palindromic SNPs with intermediate allele frequencies that would lead to ambiguous alignment to ensure that the effect estimates from exposure and outcome datasets corresponded to the exact same effect allele. All strand‐matching and direction‐harmonizing steps were automatically completed and checked through the default process of the TwoSampleMR package.

To fully validate the second core MR assumption, which requires that IVs are not associated with any predefined confounding factors, we systematically evaluated potential associations between all candidate eligible SNPs and a prespecified panel of major shared confounding factors across the mental and cardiovascular disease spectrum [18]. These predefined confounding factors included anxiety disorders and Alzheimer's disease. Any SNP that showed a statistically significant association with any of the above‐mentioned confounding factors at the conventional genome‐wide significance threshold was excluded from the final instrumental variable panel. Finally, we used the MR‐Egger regression function implemented via mr_pleiotropy_test in the R programming environment to test the third MR assumption. A nonsignificant Egger intercept with a *p‐value* greater than 0.05 indicated no substantial horizontal pleiotropy, confirming that the selected IVs influence outcome traits only through the studied exposure factors [24].

### 2.4 Statistical Analysis

Following harmonization of effect alleles across datasets for mental disorders, hypertension subtypes and related complications, three core two‐sample MR approaches were implemented to evaluate potential causal associations, namely, inverse variance weighted (IVW) analysis, MR‐Egger regression, and weighted median estimation (WME). The IVW method served as the primary analytical framework, with statistically significant causal associations defined by an IVW‐derived *p* value less than 0.05. MR‐Egger regression and WME were applied as complementary tools to corroborate the robustness of IVW results [25].

Heterogeneity across causal effect estimates from individual instrumental SNPs was quantified using the Cochran Q statistic. Assessment of heterogeneity is a critical quality control step in MR analysis, as significant heterogeneity may indicate divergent effect sizes across genetic variants, unmeasured horizontal pleiotropy, or underlying population stratification that could bias causal inference. A Cochran Q *p* value > 0.05 indicated no statistically significant heterogeneity, supporting the reliability of main results [24, 25]. When significant heterogeneity (*p* < 0.05) was detected, a random‐effects IVW model was adopted to account for between‐variant effect size variability, producing more conservative, unbiased estimates under moderate heterogeneity compared with the fixed‐effects framework, while WME results were integrated to further improve causal inference robustness [26, 27]. The three core MR methods have distinct strengths and limitations. The IVW method aggregates Wald ratio estimates from all included IVs, delivering the highest statistical power and most precise causal effect estimate when all selected genetic variants are valid, but its results are susceptible to bias from horizontal pleiotropy. The false discovery rate (FDR) correction was applied to IVW *p* values to improve data reliability (Table 2). MR‐Egger regression incorporates an unconstrained intercept term to explicitly quantify directional horizontal pleiotropy separate from the causal effect estimate [28], effectively mitigating bias from unbalanced pleiotropic effects, though it has relatively lower statistical power than IVW and relies on the instrument strength independent of direct effect assumption. A MR‐Egger intercept that shows no statistically significant deviation from zero indicates no detectable directional pleiotropy, supporting compliance with the core MR assumption. WME aggregates causal effect estimates from all genetic variants to generate a unified value, requiring more than 50% of total analytical weight to originate from valid IVs to ensure robust inference [25]. It shows strong robustness to a moderate proportion of invalid IVs but cannot provide separate quantitative estimates of horizontal pleiotropy.

**TABLE 2:** Results of IVW and WME for mental disorders and hypertension subtypes/complications.

Exposure	Outcome	IVW		Weighted median	
		*p*	FDR (*p*)	OR (95% CI)	*p*
Major depression	Hypertension	0.000	0.000	1.242 (1.132–1.363)	0.000
	Essential hypertension	0.000	0.000	1.243 (1.127–1.371)	0.000
	Secondary hypertension	0.289	0.586	0.820 (0.555–1.210)	0.316
	Pregnancy hypertension	0.307	0.586	1.063 (0.897–1.260)	0.481
	Hypertensive heart disease	0.022	0.092	1.425 (1.122–1.810)	0.004
	Hypertensive renal disease	0.660	0.794	1.046 (0.567–1.930)	0.884
	Hypertensive encephalopathy	0.206	0.481	0.395 (0.052–2.988)	0.368
Bipolar disorder	Hypertension	0.091	0.273	0.945 (0.906–0.985)	0.008
	Essential hypertension	0.040	0.140	0.940 (0.901–0.981)	0.004
	Secondary hypertension	0.905	0.940	0.913 (0.764–1.090)	0.314
	Pregnancy hypertension	0.588	0.794	1.027 (0.949–1.111)	0.511
	Hypertensive heart disease	0.004	0.021	0.924 (0.831–1.027)	0.143
	Hypertensive renal disease	0.003	0.021	1.331 (1.002–1.769)	0.049
	Hypertensive encephalopathy	0.653	0.794	0.896 (0.368–2.179)	0.809
Schizophrenia	Hypertension	0.888	0.940	0.995 (0.970–1.021)	0.700
	Essential hypertension	0.681	0.794	0.991 (0.965–1.018)	0.530
	Secondary hypertension	0.489	0.790	1.055 (0.950–1.171)	0.319
	Pregnancy hypertension	0.171	0.449	1.014 (0.967–1.063)	0.572
	Hypertensive heart disease	0.599	0.794	0.991 (0.935–1.051)	0.761
	Hypertensive renal disease	0.940	0.940	0.981 (0.839–1.147)	0.813
	Hypertensive encephalopathy	0.446	0.780	1.536 (0.932–2.529)	0.092
Hypertension	Major depression	0.008	0.044	1.020 (0.993–1.047)	0.142
	Bipolar disorder	0.840	0.845	1.049 (0.986–1.116)	0.129
	Schizophrenia	0.638	0.845	1.037 (0.990–1.086)	0.123
Essential hypertension	Major depression	0.007	0.044	1.020 (0.993–1.047)	0.142
	Bipolar disorder	0.845	0.845	1.038 (0.976–1.105)	0.235
	Schizophrenia	0.277	0.762	1.027 (0.979–1.076)	0.277
Pregnancy hypertension	Bipolar disorder	0.491	0.845	1.044 (0.956–1.139)	0.338
	Schizophrenia	0.636	0.845	1.043 (0.975–1.116)	0.218
Hypertensive heart disease	Major depression	0.265	0.762	0.999 (0.964–1.035)	0.953
	Bipolar disorder	0.818	0.845	1.005 (0.930–1.086)	0.904
	Schizophrenia	0.617	0.845	0.974 (0.920–1.033)	0.382

A comprehensive set of sensitivity analyses was conducted to validate the robustness of MR causal estimates. First, the MR‐Egger regression intercept test was used to detect directional horizontal pleiotropy. Second, leave‐one‐out analysis was performed by iteratively excluding one SNP at a time and rerunning IVW analysis, to identify outlier variants that disproportionately drive the overall causal estimate. Third, WME was adopted to generate a pleiotropy‐robust secondary causal estimate for comparison against the primary IVW result. Fourth, funnel plots were constructed to visualize the distribution of individual causal effect estimates, where a symmetric point distribution indicates no systematic bias across variants. All MR analyses were performed in R (version 4.4.0) using the TwoSampleMR package (version 0.6.18).

## 3 Results

### 3.1 MR Analysis

Following rigorous quality control procedures, we finalized eligible genetic datasets for mental disorders (defined as exposures) and various hypertension subtypes and their related complications (defined as outcomes). For the major depression trait, we harmonized its exposure dataset with each hypertension‐related outcome dataset and excluded two unaligned palindromic SNPs (rs2876520, rs4730387), resulting in 49 independent instrumental SNPs retained for analysis. Similarly, harmonizing the bipolar disorder (BD) exposure dataset with hypertension outcome datasets and removing six ineligible SNPs (rs10791849, rs11062170, rs13417268, rs1998820, rs2011302, rs62489493) yielded 49 valid instrumental SNPs. For the schizophrenia trait, harmonization with hypertension datasets and exclusion of six SNPs that failed quality control thresholds (rs13011472, rs1540840, rs1892346, rs1914399, rs1953205, rs4700418) resulted in a final panel of 153 eligible SNPs. All retained SNPs had F‐statistics > 10 (Supporting Information: Tables S1–S7), indicating strong IVs.

### 3.2 Bidirectional MR Results

Using two‐sample bidirectional MR, we systematically investigated the causal associations between mental disorders (major depression, bipolar disorder, schizophrenia) and hypertension subtypes and their related complications (hypertension, essential hypertension, secondary hypertension, pregnancy hypertension, hypertensive heart disease, hypertensive renal disease, hypertensive encephalopathy).

#### 3.2.1 Forward MR: Mental Disorders as Exposures

Forward MR analysis revealed a causal relationship between major depression and hypertension, with significant associations detected via both the IVW [odds ratio (OR) 1.228, 95% confidence interval (CI) (1.104–1.366), *p* < 0.001] and WME [OR 1.242, 95% CI (1.132–1.363), *p* < 0.001] methods. After analyzing major depression and hypertension subtypes and their complications, significant causal associations of major depression were only observed with essential hypertension [IVW: OR 1.247, 95% CI (1.117–1.393), *p* < 0.001; WME: OR 1.243, 95% CI (1.127–1.371), *p* < 0.001], as well as hypertensive heart disease [IVW: OR 1.247, 95% CI (1.033–1.505), *p* = 0.022; WME: OR 1.425, 95% CI (1.122–1.81), *p* = 0.004]. After FDR correction, the significant causal association between major depression and essential hypertension remained statistically significant (*p* < 0.05).

From a genetic perspective, there is a causal relationship between bipolar disorder and essential hypertension [IVW: OR 0.956, 95% CI (0.916–0.998), *p* = 0.040; WME: OR 0.940, 95% CI (0.901–0.981), *p* = 0.004], but it is not statistically significant after FDR testing. Nominally significant potential causal associations between bipolar disorder and both hypertensive heart disease and hypertensive renal disease were also observed (*p* < 0.05). As presented in Figure 2, no significant causal association was detected between schizophrenia and any subtype or complication of hypertension.

**FIGURE 2:**
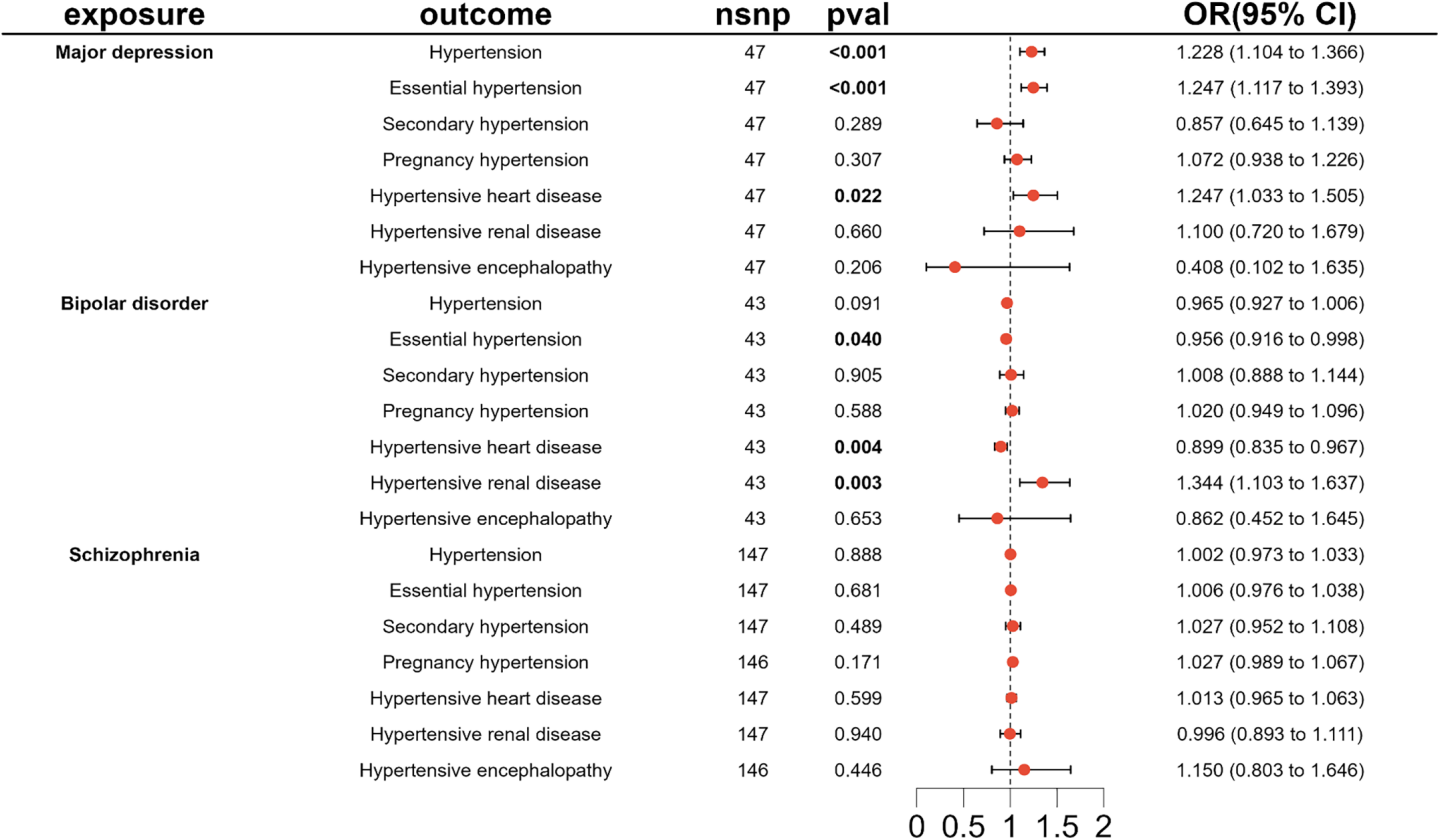
IVW results of mental disorders and hypertension subtypes/complications.

#### 3.2.2 Reverse MR: Hypertension Subtype/Complication as Exposures

Reverse MR analysis validated a causal relationship between hypertension and major depression, with a significant association via the IVW method (OR 1.028, 95% CI (1.007–1.050), *p* = 0.008). After detailed stratified analyses across different hypertension subtypes and their complications, only essential hypertension was found to be associated with an increased risk of major depression (IVW: OR 1.031, 95% CI (1.009–1.054), *p* = 0.007), and this association remained statistically significant after FDR correction (*p* < 0.05). Notably, no causal associations were observed between any hypertension subtype or complication and bipolar disorder or schizophrenia (Figure 3).

**FIGURE 3:**
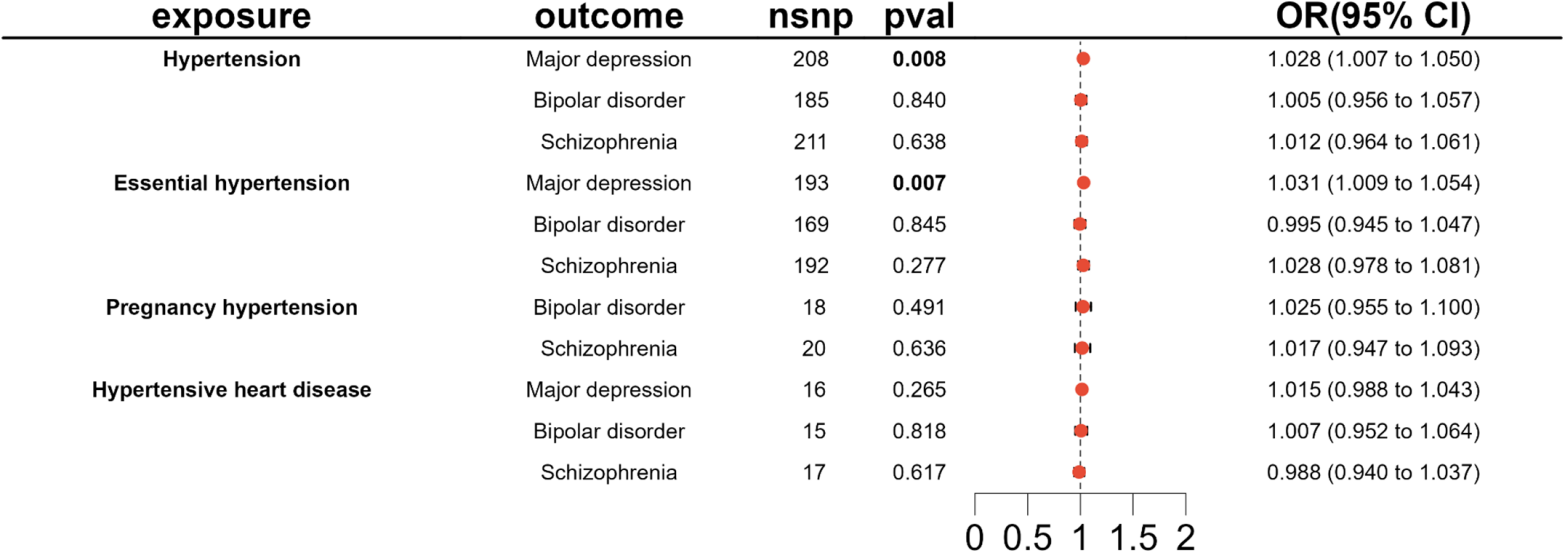
IVW results of hypertension subtypes/complications and mental disorders.

### 3.3 Sensitivity Analysis

To systematically assess the robustness of our causal estimates, we conducted a series of sensitivity analyses, starting with Cochran's Q test to evaluate heterogeneity in IVW and MR‐Egger regression results (Table 3). Among statistically significant MR associations (positive causal links), Cochran's Q test yielded *p* values > 0.05 for bipolar disorder and hypertensive renal disease, suggesting no substantial heterogeneity. However, *p* values < 0.05 were observed for other significant associations, indicating the presence of heterogeneity. To mitigate the impact of this heterogeneity and improve result reliability, a random‐effects model was applied to all IVW analyses involving heterogeneous datasets. WME results were additionally reported as supplementary data (Table 2) to complement the primary IVW findings. We further performed leave‐one‐out sensitivity analyses and generated funnel plots to assess bias. Funnel plot symmetry suggested a low risk of publication bias or small‐study effects. Leave‐one‐out analysis (removing one SNP at a time) revealed no individual SNP significantly altered overall MR estimates, confirming the robustness of our results. Scatter plots were constructed to visualize the relationship between SNP effects on exposures and outcomes, while forest plots displayed individual SNP estimates (via Wald ratio) as horizontal lines. Notably, MR‐Egger intercept tests revealed intercept values very close to 0 (*p* > 0.05 for all endpoints), indicating no significant horizontal pleiotropy. Leave‐one‐out plots, funnel plots, scatter plots, and forest plots are provided in the Supporting Information (Figures S1–S32).

**TABLE 3:** Horizontal pleiotropy in mental disorders and hypertension subtypes/complications.

Exposure	Outcome	Heterogeneity test				Pleiotropy test		
		IVW		MR‐Egger		MR‐Egger		
		Q	Q_pval	Q	Q_pval	Intercept	Se	*p*
Major depression	Hypertension	173.129	0.000	173.093	0.000	0.001	0.010	0.923
	Essential hypertension	168.066	0.000	167.857	0.000	0.002	0.010	0.814
	Secondary hypertension	50.433	0.303	49.620	0.294	0.022	0.026	0.395
	Pregnancy hypertension	64.511	0.037	63.562	0.035	−0.010	0.012	0.417
	Hypertensive heart disease	68.867	0.016	68.413	0.014	0.009	0.017	0.587
	Hypertensive renal disease	43.691	0.569	41.696	0.613	0.053	0.038	0.165
	Hypertensive encephalopathy	39.457	0.741	39.276	0.712	0.053	0.124	0.672
Bipolar disorder	Hypertension	107.289	0.000	102.769	0.000	−0.009	0.007	0.187
	Essential hypertension	107.111	0.000	103.659	0.000	−0.009	0.007	0.249
	Secondary hypertension	39.794	0.568	39.495	0.538	0.012	0.022	0.588
	Pregnancy hypertension	77.928	0.001	72.319	0.002	−0.022	0.012	0.082
	Hypertensive heart disease	43.966	0.388	43.964	0.347	0.000	0.013	0.975
	Hypertensive renal disease	40.130	0.553	39.514	0.537	−0.027	0.034	0.437
	Hypertensive encephalopathy	24.724	0.984	24.598	0.980	0.040	0.111	0.725
Schizophrenia	Hypertension	649.567	0.000	649.506	0.000	0.000	0.004	0.907
	Essential hypertension	618.400	0.000	617.683	0.000	0.002	0.004	0.682
	Secondary hypertension	169.723	0.087	169.605	0.079	0.003	0.011	0.751
	Pregnancy hypertension	243.723	0.000	243.567	0.000	−0.002	0.005	0.762
	Hypertensive heart disease	217.998	0.000	216.716	0.000	0.006	0.007	0.356
	Hypertensive renal disease	133.894	0.755	133.567	0.742	0.009	0.015	0.568
	Hypertensive encephalopathy	116.855	0.959	116.603	0.954	−0.025	0.050	0.616
Hypertension	Major depression	343.138	0.000	341.853	0.000	0.001	0.001	0.380
	Bipolar disorder	332.301	0.000	332.280	0.000	0.000	0.003	0.915
	Schizophrenia	612.260	0.000	608.979	0.000	−0.003	0.003	0.290
Essential hypertension	Major depression	361.505	0.000	359.179	0.000	0.002	0.002	0.268
	Bipolar disorder	303.201	0.000	297.914	0.000	−0.006	0.003	0.087
	Schizophrenia	591.408	0.000	590.635	0.000	−0.002	0.003	0.619
Pregnancy hypertension	Bipolar disorder	23.834	0.124	20.439	0.201	−0.018	0.011	0.123
	Schizophrenia	47.393	0.000	44.913	0.000	−0.012	0.012	0.332
Hypertensive heart disease	Major depression	17.973	0.264	17.957	0.209	−0.001	0.006	0.912
	Bipolar disorder	12.903	0.534	12.416	0.494	0.008	0.012	0.498
	Schizophrenia	22.543	0.127	22.040	0.107	−0.007	0.011	0.567

## 4 Discussion

Through bidirectional MR analysis, this study identified a potential genetic causal association between major depression and essential hypertension. Forward MR analysis showed that the genetic susceptibility to major depression significantly increased the risk of essential hypertension (IVW: OR = 1.247), and the causal estimate generated by the weighted median approach was consistent with the primary IVW result. Reverse MR analysis also indicated that essential hypertension exerted a reverse causal effect on the risk of major depression (OR = 1.031). Our bidirectional MR analysis revealed a robust, genetically supported causal relationship between major depression and essential hypertension, filling a crucial evidence gap in the understanding of the pathophysiological mechanisms of their interrelationship beyond traditional observational associations and offering new insights into the shared molecular pathways underlying their frequent clinical comorbidity.

More than 90% of all hypertension cases are ultimately classified as essential hypertension. Multiple biological and behavioral mechanisms may explain the observed association between depression and hypertension. In the pathophysiological pathway leading from major depression to elevated hypertension risk, the pathological cascade is initiated by persistent dysregulation of the core neuroendocrine axis [29]. A long‐term depressive state continuously activates the hypothalamic–pituitary–adrenal axis (HPA axis), leading to elevated levels of catecholamines and cortisol in the circulation [29, 30]. These neurohormonal changes directly trigger peripheral vasoconstriction, renal sodium and water retention, and gradually increase the basal pressure–set blood point, ultimately resulting in hypertension [31, 32]. Prior studies have reported that genetic variants in the *BDNF*, *CREB1*, *GNAS*, and *POMC* genes within the corticotropin‐releasing hormone (CRH) signaling pathway are associated with major depression and hypertension [33]. These genes belong to the stress‐responsive genome, which can regulate the activity of the HPA axis by activating it and, simultaneously or through mediators, increase the risk of cardiometabolic and mood disorders [34, 35, 36, 37]. At the molecular signaling level, gene variants enriched in individuals with depression can disrupt energy homeostasis mediated by the CRH signaling pathway and AMP‐activated protein kinase, further exacerbating sympathetic overactivity and inducing chronic low‐grade systemic inflammation [33, 38, 39, 40]. This inflammatory microenvironment can damage vascular endothelial function, accelerate the progression of atherosclerosis, and form a pro‐hypertensive vascular phenotype [33]. Paton et al. [41] reported elevated levels of inflammatory cells and pro‐inflammatory cytokines in the brain. These inflammatory mediators can impair the blood pressure regulatory function of the central autonomic nervous system (including the hypothalamic‐sympathetic axis), thereby further accelerating the development of hypertension [41]. The superposition of these biological effects, together with unhealthy behaviors that are continuously associated with major depression, such as high‐sodium diet, reduced physical activity, and substance abuse, act synergistically to increase the pathological stress load and ultimately accelerate the onset of essential hypertension.

Comorbidity of major depression and essential hypertension also involves autonomic nervous system dysfunction, creating a bidirectional pathological cycle [42, 43, 44]. Patients with depression exhibit abnormal autonomic nervous system activity, characterized by enhanced sympathetic nerve activity (i.e., sympathetic hyperactivity) and reduced vagal tone [42]. This dysregulation, evidenced by elevated plasma norepinephrine levels and increased concentrations of its metabolite, 3‐methoxy‐4‐hydroxyphenylethanol [45, 46, 47], parallels the comparable sympathetic hyperactivity observed in patients with essential hypertension. Maes et al. [48] found that sleep disorders, a typical clinical symptom in patients with depression, were positively correlated with catecholamine (norepinephrine, epinephrine) excretion, implicating sympathetic overactivation as a key driver of depression‐induced hypertension. Conversely, chronic stress in hypertensive patients worsens emotional dysfunction, further stimulating sympathetic activity and perpetuating a vicious cycle [49]. Reduced vagal tone is an independent predictor of new‐onset hypertension [50]. Long‐term stress‐induced overactivation of the sympathetic nervous system and HPA axis, combined with reduced vagal tone, increases the risk of early‐onset coronary events and further exacerbates the overall comorbidity burden [51].

Dysregulation of serotonin and dopamine receptor‐mediated neurotransmission represents another key mechanism underlying the comorbidity of mental disorders and essential hypertension, directly linking central emotional regulation processes to peripheral metabolic and cardiovascular processes [52]. In the serotonin pathway, genetic variants in *SLC18A1* [53] and *HTR1A* [54] disrupt serotonergic neurotransmission, contributing to depression while inducing insulin resistance and elevated blood pressure via disrupting insulin‐serotonin crosstalk in β‐islet cells [33]. In the dopamine pathway, genetic variants in *GNAS* [55, 56] and *PPP1R1B* [57, 58] impair dopamine signaling. This pathway not only modulates brain functions including emotion and motivation but also regulates peripheral processes in the kidneys and cardiovascular system (such as vascular contraction), thereby directly linking the pathogenesis of emotional disorders to the development of essential hypertension [33], as depicted in Figure 4.

**FIGURE 4:**
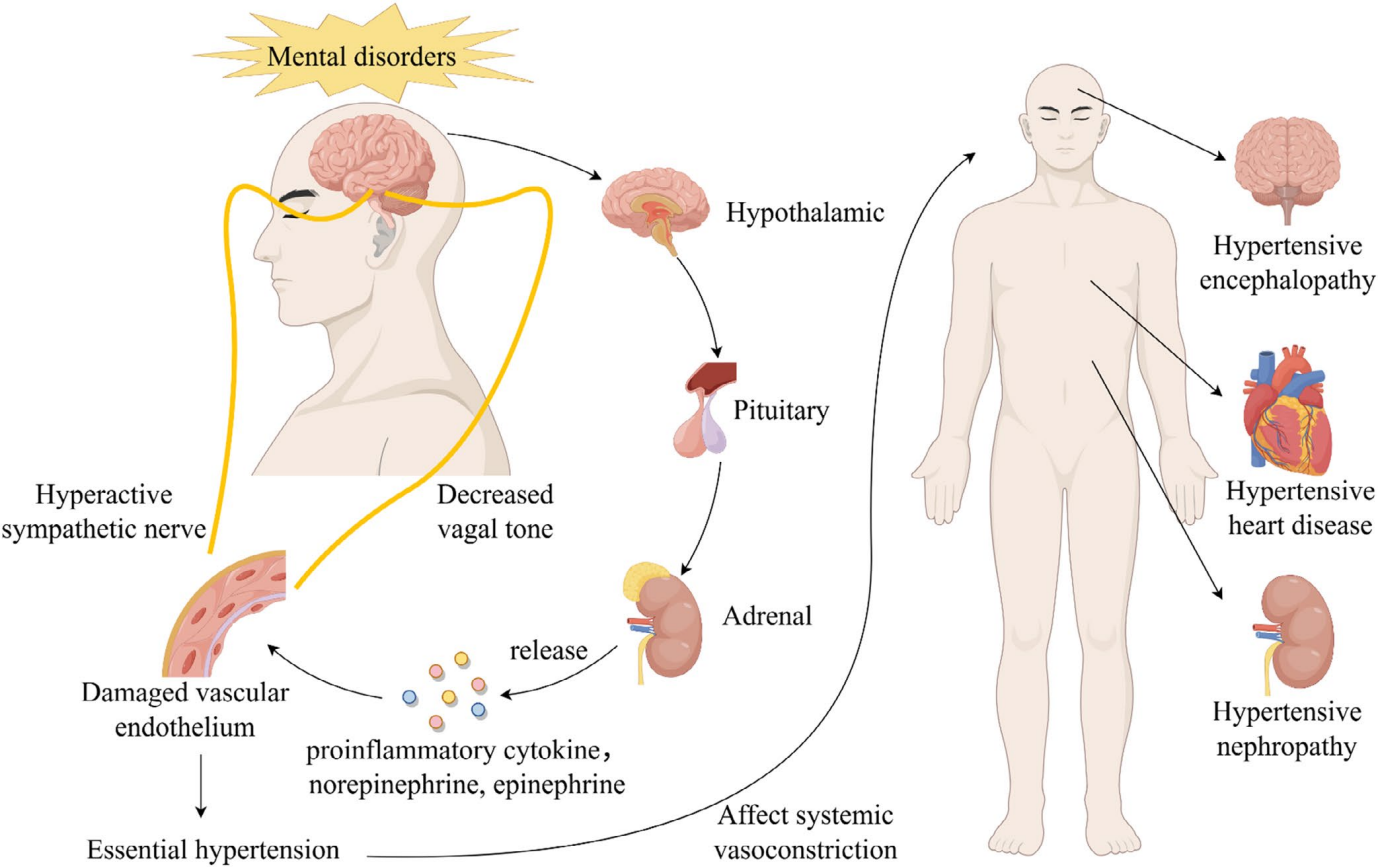
The mechanism of the association between mental disorders and essential hypertension.

In the reverse process where essential hypertension increases the risk of major depression, the pathological mechanism progresses through three interrelated stages. First, long‐term elevated arterial blood pressure triggers irreversible damage to the cerebral microvasculature, manifested as extensive white matter ischemic lesions, cerebral microinfarcts, and cerebral microhemorrhages [59, 60]. These structural abnormalities directly disrupt the integrity of the prefrontal and limbic system neural circuits, which are responsible for emotional processing, reward regulation, and stress response [42, 59]. Second, a prominent feature of persistent hypertension is the gradual desensitization of sympathetic receptors, which distorts the brainstem's processing of interoceptive signals and affects functions related to emotional perception and pain regulation, thus breaking the normal regulatory feedback loop that maintains emotional stability [59, 61, 62, 63]. This vascular‐driven neurological dysfunction further interferes with the central monoamine neurotransmitter metabolism, reducing the bioavailability of serotonin and norepinephrine, both of which are crucial for maintaining a normal emotional state [33, 42]. Third, the psychological labeling effect arising after the formal diagnosis of hypertension, the long‐term treatment burden, and the shared genetic susceptibility loci including *BDNF*, *CREB1*, and *GNAS* together lead to additional emotional distress [33, 64]. All these factors interact to form a self‐reinforcing pathological cycle: hypertension‐associated neurobiological changes exacerbate the severity of depressive symptoms, and these depressive symptoms in turn further interfere with long‐term optimal blood pressure control.

Our research results indicate that no statistically significant causal relationship exists between schizophrenia and any of the included hypertension subtypes and their complications. While bipolar disorder shows nominally significant associations with essential hypertension and hypertensive heart disease, the corresponding odds ratios are less than 1, and no statistically significant causal association between bipolar disorder and essential hypertension remains detectable after FDR correction. Previous work has reported that a subset of genes strongly associated with bipolar disorder, such as *ANK3* and *CACNA1C*, may simultaneously modulate bipolar disorder pathogenesis and its related systemic inflammatory factors, indirectly reducing the risk of essential hypertension and masking potential harmful cardiovascular effects of bipolar disorder [65, 66, 67, 68], ultimately leading to reduced incidence of hypertensive heart disease. Bipolar disorder is classified into Type I and Type II subtypes [67], and our inability to stratify analyses by these two distinct subtypes may introduce residual analytical bias. Additionally, existing evidence suggests that lithium carbonate treatment for bipolar disorder exerts a blood pressure‐lowering effect by inhibiting the release of norepinephrine and epinephrine [69].

We performed a bidirectional MR design to systematically investigate the genetic causal associations between three major psychiatric disorders and distinct subtypes of hypertension as well as their corresponding complications. This approach effectively mitigates common confounding bias and reverse causality inherent in conventional observational studies, eliminates the requirement for prolonged clinical follow‐up, and confers notable advantages in cost efficiency and ethical compliance. It therefore serves as a robust preclinical analytical tool for exploring the etiology of cardio‐psychiatric comorbidity. Translating the study findings into clinical practice, we recommend routine blood pressure and target organ function monitoring for patients with major depression, along with dynamic surveillance of HPA axis activity to facilitate timely adjustment of therapeutic regimens. For individuals with bipolar disorder, targeted awareness of hypertension‐related kidney injury risk and regular renal function screening are advised. Several limitations of this study should be explicitly acknowledged. First, a subset of genetic variants associated with psychiatric disorders exhibits relatively small effect sizes on blood pressure phenotypes, which may reduce statistical power and increase the likelihood of false‐negative results. Existing dataset constraints also prevented sufficient statistical power for formal analysis of causal associations between secondary hypertension and the three psychiatric disorders. Second, all GWAS summary statistics included in the current analysis were derived from populations of European ancestry. This ancestral specificity inherently restricts the direct generalizability of the observed genetic causal associations to non‐European populations, including but not limited to East Asian, African, and admixed groups [70]. Third, the present study only included three broad categories of core psychiatric conditions, without incorporating distinct subtypes such as perinatal depression and bipolar disorder Type I. Stratified analyses for schizophrenia based on age at onset, illness severity, and treatment response were also not performed. Given that MR inherently estimates the long‐term effects of lifelong genetic exposure, while the peak incidence of schizophrenia occurs in early adulthood and the clinical manifestation of essential hypertension requires decades of subclinical pathological accumulation, such temporal mismatch may result in the genetic susceptibility effect of schizophrenia being masked by stronger effects from conventional cardiovascular risk factors before it can fully contribute to the pathogenesis of hypertension. This scenario provides a plausible explanation for the absence of statistically significant causal associations between schizophrenia and hypertension in the overall population, though subtle potential associations in specific clinical subgroups cannot be entirely excluded. The mediating effects underlying complex pleiotropic pathways remain to be fully elucidated, which will be prioritized in subsequent research. Future investigations will expand trans‐ancestry sample sizes to improve the detection capability for weak‐effect genetic loci, with deliberate enrollment of Asian population cohorts. Subsequent studies will also systematically characterize the subtype‐specific associations between psychiatric disorders and hypertension and integrate multi‐omics analyses with experimental validations to dissect the underlying pathophysiological mechanisms, thereby generating more rigorous evidence to support precision clinical management and public health policy formulation.

## 5 Conclusion

We conducted a bidirectional MR study to explore the potential genetic associations between major depression, bipolar disorder, schizophrenia, and distinct hypertension subtypes and their related complications. The results of the study indicate that the genetic predisposition to major depression is causally associated with an increased risk of essential hypertension. Similarly, the genetic susceptibility to essential hypertension also shows a slight association with the risk of depression. Although these results support a plausible bidirectional causal relationship, they reflect long‐term genetically predicted effects rather than acute short‐term clinical outcomes. Previous studies have proposed that potential biological pathways such as abnormal HPA axis and autonomic nervous system dysregulation may explain the relationship between major depression and hypertension. However, the present study did not directly validate these specific mechanistic pathways, so such interpretations should be regarded as hypothetical directions for future independent research. Further targeted experimental and large‐scale prospective clinical studies are needed to validate these causal associations and fully elucidate their underlying pathophysiological mechanisms.

## Funding

The authors have nothing to report.

## Ethics Statement

The authors have nothing to report.

## Consent

The authors have nothing to report.

## Conflicts of Interest

The authors declare no conflicts of interest.

## Supporting information


**Figure S1:** The causal effect of major depression on hypertension.
**Figure S2:** The causal effect of major depression on essential hypertension.
**Figure S3:** The causal effect of major depression on secondary hypertension.
**Figure S4:** The causal effect of major depression on pregnancy hypertension.
**Figure S5:** The causal effect of major depression on hypertensive heart disease.
**Figure S6:** The causal effect of major depression on hypertensive encephalopathy.
**Figure S7:** The causal effect of major depression on hypertensive renal disease.
**Figure S8:** The causal effect of bipolar disorder on hypertension.
**Figure S9:** The causal effect of bipolar disorder on essential hypertension.
**Figure S10:** The causal effect of bipolar disorder on secondary hypertension.
**Figure S11:** The causal effect of bipolar disorder on C hypertension.
**Figure S12:** The causal effect of bipolar disorder on hypertensive heart disease.
**Figure S13:** The causal effect of bipolar disorder on hypertensive encephalopathy.
**Figure S14:** The causal effect of bipolar disorder on hypertensive renal disease.
**Figure S15:** The causal effect of schizophrenia on hypertension.
**Figure S16:** The causal effect of schizophrenia on essential hypertension.
**Figure S17:** The causal effect of schizophrenia on secondary hypertension.
**Figure S18:** The causal effect of schizophrenia on pregnancy hypertension.
**Figure S19:** The causal effect of schizophrenia on hypertensive heart disease.
**Figure S20:** The causal effect of schizophrenia on hypertensive encephalopathy.
**Figure S21:** The causal effect of schizophrenia on hypertensive renal disease.
**Figure S22:** The causal effect of hypertension on major depression.
**Figure S23:** The causal effect of hypertension on bipolar disorder.
**Figure S24:** The causal effect of hypertension on schizophrenia.
**Figure S25:** The causal effect of essential hypertension on major depression.
**Figure S26:** The causal effect of essential hypertension on bipolar disorder.
**Figure S27:** The causal effect of essential hypertension on schizophrenia.
**Figure S28:** The causal effect of pregnancy hypertension on bipolar disorder.
**Figure S29:** The causal effect of pregnancy hypertension on schizophrenia.
**Figure S30:** The causal effect of hypertensive heart disease on major depression.
**Figure S31:** The causal effect of hypertensive heart disease on bipolar disorder.
**Figure S32:** The causal effect of hypertensive heart disease on schizophrenia.
**Table S1:** 50 SNPs associated with depression.
**Table S2:** 52 SNPs associated with bipolar disorder.
**Table S3:** 154 SNPs associated with schizophrenia.
**Table S4:** 270 SNPs associated with hypertension.
**Table S5:** 232 SNPs associated with essential hypertension.
**Table S6:** 28 SNPs associated with pregnancy hypertension.
**Table S7:** 19 SNPs associated with hypertensive heart disease.

## Data Availability

The dataset presented herein can be accessed via an online database, whose name is provided in the article. The original contributions presented in the study are included in the article/Supporting Information; further inquiries can be directed to the corresponding authors.
