# Supplementary material for: Investigating Causal Relationships Between Major Mental Disorders and Hypertension Subtypes and Their Complications: A Mendelian Randomization Study
Source: Genes Brain Behav. 2026 Nov 8;25(6):e70078. doi: 10.1111/gbb.70078 (PMC13745640; doi:10.1111/gbb.70078)
Supplement: Supplementary file 1 [file GBB-25-e70078-s001.docx]

**Figure S1: The causal effect of** **Major depression on Hypertension**

**Figure S2: The causal effect of** **Major depression on Essential hypertension**

**Figure S3: The causal effect of** **Major depression on** **Secondary hypertension**

**Figure S4:** **The causal effect of** **Major depression on Pregnancy hypertension**

**Figure S5: The causal effect of** **Major depression on Hypertensive heart disease**

**Figure S6:** **The causal effect of** **Major depression on Hypertensive encephalopathy**

**Figure S7: The causal effect of** **Major depression on Hypertensive renal disease**

**Figure S8: The causal effect of** **Bipolar disorder on Hypertension**

**Figure S9: The causal effect of** **Bipolar disorder on Essential hypertension**

**Figure S10: The causal effect of** **Bipolar disorder on Secondary hypertension**

**Figure S11: The causal effect of** **Bipolar disorder on Pregnancy hypertension**

**Figure S12: The causal effect of** **Bipolar disorder on Hypertensive heart disease**

**Figure S13: The causal effect of** **Bipolar disorder on Hypertensive encephalopathy**

**Figure S14: The causal effect of** **Bipolar disorder on Hypertensive renal disease**

**Figure S15: The causal effect of** **Schizophrenia on Hypertension**

**Figure S16: The causal effect of** **Schizophrenia on Essential hypertension**

**Figure S17: The causal effect of** **Schizophrenia on Secondary hypertension**

**Figure S18: The causal effect of** **Schizophrenia on Pregnancy hypertension**

**Figure S19: The causal effect of** **Schizophrenia on Hypertensive heart disease**

**Figure S20: The causal effect of** **Schizophrenia on Hypertensive encephalopathy**

**Figure S21: The causal effect of** **Schizophrenia on Hypertensive renal disease**

**Figure S22: The causal effect of Hypertension on Major depression**

**Figure S23: The causal effect of Hypertension on Bipolar disorder**

**Figure S24: The causal effect of Hypertension on Schizophrenia**

**Figure S25: The causal effect of Essential hypertension on Major depression**

**Figure S26: The causal effect of Essential hypertension on Bipolar disorder**

**Figure S27: The causal effect of Essential hypertension on Schizophrenia**

**Figure S28: The causal effect of** **Pregnancy hypertension on Bipolar disorder**

**Figure S29: The causal effect of** **Pregnancy hypertension on Schizophrenia**

**Figure S30: The causal effect of Hypertensive heart disease on Major depression**

**Figure S31: The causal effect of Hypertensive heart disease on Bipolar disorder**

**Figure S32: The causal effect of Hypertensive heart disease on Schizophrenia**

**Table S1. 50 SNPs associated with depression.**

| SNP | Effect  allele | Other allele | Effect allele  frequency | Beta | Se | Pval | Sample  size | F |
| --- | --- | --- | --- | --- | --- | --- | --- | --- |
| rs4141983 | C | T | 0.326 | -0.026 | 0.005 | 9.69E-09 | 500,199 | 32.938 |
| rs354155 | C | G | 0.0923 | -0.045 | 0.008 | 1.75E-09 | 500,199 | 35.840 |
| rs7551758 | G | T | 0.5329 | 0.028 | 0.004 | 5.11E-11 | 500,199 | 43.315 |
| rs7538938 | C | T | 0.5599 | 0.025 | 0.004 | 7.29E-09 | 500,199 | 34.073 |
| rs2568958 | A | G | 0.6042 | 0.038 | 0.004 | 2.90E-18 | 500,199 | 75.374 |
| rs10913112 | T | C | 0.378 | -0.026 | 0.005 | 4.53E-09 | 500,199 | 33.898 |
| rs17641524 | T | C | 0.2101 | -0.030 | 0.005 | 1.50E-08 | 500,199 | 32.040 |
| rs2111592 | A | G | 0.3141 | 0.026 | 0.005 | 1.35E-08 | 500,199 | 32.689 |
| rs72948506 | A | G | 0.2975 | 0.027 | 0.005 | 1.71E-08 | 500,199 | 31.790 |
| rs9831648 | T | G | 0.7739 | -0.029 | 0.005 | 1.59E-08 | 500,199 | 31.533 |
| rs843812 | A | G | 0.4117 | 0.025 | 0.004 | 1.41E-08 | 500,199 | 31.769 |
| rs76954012 | A | T | 0.0931 | 0.041 | 0.007 | 2.41E-08 | 500,199 | 30.998 |
| rs66511648 | C | T | 0.284 | 0.030 | 0.005 | 6.03E-10 | 500,199 | 38.285 |
| rs699927 | G | T | 0.4171 | 0.024 | 0.004 | 3.79E-08 | 500,199 | 29.752 |
| rs247910 | G | A | 0.457 | 0.024 | 0.004 | 4.71E-08 | 500,199 | 30.378 |
| rs30266 | A | G | 0.3271 | 0.037 | 0.005 | 1.43E-15 | 500,199 | 63.306 |
| rs7725715 | A | G | 0.5343 | 0.029 | 0.004 | 1.61E-11 | 500,199 | 45.484 |
| rs150186873 | C | A | 0.0327 | 0.070 | 0.012 | 4.51E-09 | 500,199 | 34.418 |
| rs2232423 | G | A | 0.1056 | -0.062 | 0.007 | 1.14E-18 | 500,199 | 78.449 |
| rs2214123 | G | A | 0.6466 | -0.026 | 0.005 | 8.56E-09 | 500,199 | 33.640 |
| rs2876520 | G | C | 0.4688 | 0.026 | 0.004 | 2.24E-09 | 500,199 | 36.560 |
| rs9364755 | G | A | 0.2262 | 0.028 | 0.005 | 3.49E-08 | 500,199 | 30.792 |
| rs10235664 | C | T | 0.2529 | -0.027 | 0.005 | 4.68E-08 | 500,199 | 30.362 |
| rs3807865 | A | G | 0.4105 | 0.031 | 0.004 | 1.09E-12 | 500,199 | 49.638 |
| rs59082935 | T | C | 0.1342 | 0.036 | 0.007 | 3.07E-08 | 500,199 | 30.250 |
| rs2522831 | C | T | 0.4739 | 0.024 | 0.004 | 2.11E-08 | 500,199 | 31.152 |
| rs4730387 | A | T | 0.4659 | 0.024 | 0.004 | 4.12E-08 | 500,199 | 30.635 |
| rs150346963 | T | C | 0.4118 | 0.028 | 0.004 | 1.16E-10 | 500,199 | 41.368 |
| rs1931388 | G | A | 0.4042 | -0.030 | 0.004 | 1.68E-11 | 500,199 | 44.951 |
| rs59283172 | A | G | 0.1081 | -0.039 | 0.007 | 2.41E-08 | 500,199 | 31.041 |
| rs62535714 | A | G | 0.1639 | 0.034 | 0.006 | 4.69E-09 | 500,199 | 34.162 |
| rs2418449 | C | T | 0.281 | -0.028 | 0.005 | 4.25E-09 | 500,199 | 34.271 |
| rs1021363 | G | A | 0.6434 | -0.030 | 0.005 | 2.29E-11 | 500,199 | 44.444 |
| rs198457 | T | C | 0.1886 | -0.032 | 0.006 | 1.90E-08 | 500,199 | 31.641 |
| rs4497414 | C | T | 0.44 | 0.029 | 0.004 | 2.93E-11 | 500,199 | 43.740 |
| rs4936276 | C | G | 0.622 | 0.028 | 0.004 | 3.57E-10 | 500,199 | 39.919 |
| rs61914045 | A | G | 0.2034 | 0.031 | 0.005 | 7.96E-09 | 500,199 | 32.744 |
| rs9529218 | T | C | 0.2031 | -0.034 | 0.005 | 2.23E-10 | 500,199 | 39.643 |
| rs9536381 | T | C | 0.3259 | 0.026 | 0.005 | 2.62E-08 | 500,199 | 30.730 |
| rs508502 | T | C | 0.2992 | -0.026 | 0.005 | 3.56E-08 | 500,199 | 30.250 |
| rs1950829 | G | A | 0.5173 | -0.030 | 0.004 | 4.74E-12 | 500,199 | 47.706 |
| rs7152906 | C | T | 0.5196 | 0.026 | 0.004 | 1.87E-09 | 500,199 | 36.000 |
| rs754287 | A | T | 0.3664 | -0.029 | 0.005 | 1.31E-10 | 500,199 | 41.245 |
| rs28541419 | G | C | 0.2308 | -0.029 | 0.005 | 1.76E-08 | 500,199 | 31.533 |
| rs12919291 | C | G | 0.1884 | 0.033 | 0.006 | 3.09E-09 | 500,199 | 35.348 |
| rs4799949 | T | C | 0.6684 | -0.029 | 0.005 | 1.40E-10 | 500,199 | 40.295 |
| rs1367635 | C | T | 0.5148 | 0.025 | 0.004 | 4.35E-09 | 500,199 | 34.618 |
| rs12967143 | C | G | 0.7012 | -0.035 | 0.005 | 2.53E-13 | 500,199 | 53.882 |
| rs7241572 | A | G | 0.2047 | 0.032 | 0.005 | 2.43E-09 | 500,199 | 35.778 |
| rs13037326 | T | C | 0.2597 | 0.031 | 0.005 | 2.40E-10 | 500,199 | 40.025 |

**Table S2. 52 SNPs associated with bipolar disorder.**

| SNP | Effect  allele | Other allele | Beta | Se | Pval | Sample  size | F |
| --- | --- | --- | --- | --- | --- | --- | --- |
| rs2126180 | A | G | 0.057 | 0.009 | 1.62E-09 | 413,466 | 36.258 |
| rs10737496 | T | C | -0.054 | 0.009 | 7.17E-09 | 413,466 | 33.251 |
| rs4619651 | A | G | -0.066 | 0.010 | 4.78E-11 | 413,466 | 42.827 |
| rs17183814 | A | G | -0.103 | 0.019 | 2.68E-08 | 413,466 | 30.937 |
| rs13417268 | G | C | -0.062 | 0.011 | 2.05E-08 | 413,466 | 31.564 |
| rs2011302 | A | T | 0.053 | 0.010 | 4.25E-08 | 413,466 | 29.748 |
| rs9834970 | C | T | 0.083 | 0.009 | 6.63E-19 | 413,466 | 79.653 |
| rs2336147 | C | T | -0.068 | 0.009 | 3.61E-13 | 413,466 | 52.986 |
| rs6806239 | G | T | -0.066 | 0.012 | 2.64E-08 | 413,466 | 30.851 |
| rs696366 | A | C | -0.052 | 0.009 | 4.46E-08 | 413,466 | 30.128 |
| rs112481526 | G | A | 0.063 | 0.011 | 1.86E-09 | 413,466 | 36.114 |
| rs28565152 | A | G | 0.067 | 0.011 | 1.96E-09 | 413,466 | 35.895 |
| rs7707252 | G | A | 0.057 | 0.010 | 3.64E-08 | 413,466 | 30.245 |
| rs6887473 | A | G | -0.060 | 0.011 | 8.81E-09 | 413,466 | 32.985 |
| rs10043984 | T | C | 0.059 | 0.011 | 3.71E-08 | 413,466 | 30.153 |
| rs10866641 | C | T | -0.063 | 0.009 | 2.79E-11 | 413,466 | 44.349 |
| rs13195401 | T | G | -0.136 | 0.018 | 6.98E-15 | 413,466 | 60.308 |
| rs41315395 | A | C | 0.072 | 0.013 | 1.48E-08 | 413,466 | 32.055 |
| rs1487445 | T | C | 0.074 | 0.009 | 1.48E-15 | 413,466 | 63.649 |
| rs10455979 | G | C | 0.056 | 0.010 | 4.22E-09 | 413,466 | 34.250 |
| rs12668848 | A | G | -0.057 | 0.010 | 1.90E-09 | 413,466 | 36.005 |
| rs113779084 | A | G | 0.075 | 0.010 | 1.42E-13 | 413,466 | 54.789 |
| rs6954854 | A | G | -0.058 | 0.009 | 5.94E-10 | 413,466 | 38.463 |
| rs11764361 | G | A | -0.061 | 0.010 | 3.47E-09 | 413,466 | 34.968 |
| rs6946056 | C | A | 0.053 | 0.010 | 3.66E-08 | 413,466 | 30.081 |
| rs10255167 | A | G | 0.066 | 0.012 | 1.60E-08 | 413,466 | 31.661 |
| rs62489493 | G | C | 0.090 | 0.014 | 2.64E-11 | 413,466 | 44.144 |
| rs2953928 | A | G | 0.116 | 0.020 | 6.25E-09 | 413,466 | 33.696 |
| rs6992333 | G | A | 0.060 | 0.010 | 1.62E-09 | 413,466 | 36.236 |
| rs62581014 | T | C | 0.065 | 0.012 | 2.77E-08 | 413,466 | 31.055 |
| rs1998820 | A | T | -0.084 | 0.015 | 4.10E-08 | 413,466 | 30.321 |
| rs10994415 | C | T | 0.118 | 0.017 | 1.14E-11 | 413,466 | 46.066 |
| rs2273738 | T | C | 0.092 | 0.014 | 1.63E-11 | 413,466 | 45.462 |
| rs174592 | G | A | 0.072 | 0.010 | 9.92E-14 | 413,466 | 55.098 |
| rs10791849 | A | T | -0.069 | 0.012 | 9.89E-09 | 413,466 | 32.892 |
| rs12575685 | A | G | 0.065 | 0.010 | 1.24E-10 | 413,466 | 41.673 |
| rs7108878 | G | T | 0.081 | 0.015 | 3.61E-08 | 413,466 | 30.365 |
| rs11062170 | C | G | 0.078 | 0.010 | 1.87E-15 | 413,466 | 63.181 |
| rs35306827 | A | G | -0.066 | 0.011 | 3.56E-09 | 413,466 | 34.621 |
| rs2693698 | G | A | 0.053 | 0.009 | 1.96E-08 | 413,466 | 31.790 |
| rs35958438 | A | G | -0.064 | 0.012 | 3.83E-08 | 413,466 | 30.113 |
| rs4447398 | C | A | -0.082 | 0.014 | 2.61E-09 | 413,466 | 35.478 |
| rs748455 | C | T | -0.067 | 0.010 | 5.01E-11 | 413,466 | 42.947 |
| rs28455634 | A | G | -0.063 | 0.010 | 2.63E-10 | 413,466 | 40.235 |
| rs7201930 | C | T | 0.059 | 0.010 | 1.89E-08 | 413,466 | 31.753 |
| rs4790841 | T | C | 0.073 | 0.013 | 3.14E-08 | 413,466 | 30.504 |
| rs61554907 | T | G | 0.087 | 0.015 | 1.64E-08 | 413,466 | 31.769 |
| rs228768 | T | G | -0.064 | 0.010 | 2.83E-10 | 413,466 | 39.864 |
| rs6104027 | G | A | -0.060 | 0.010 | 1.93E-10 | 413,466 | 40.295 |
| rs237460 | T | C | 0.055 | 0.009 | 4.25E-09 | 413,466 | 34.611 |
| rs13044225 | G | A | 0.055 | 0.010 | 8.50E-09 | 413,466 | 33.152 |
| rs5758064 | C | T | -0.052 | 0.009 | 2.01E-08 | 413,466 | 31.750 |

**Table S3. 154 SNPs associated with schizophrenia.**

| SNP | Effect  allele | Other allele | Effect allele  frequency | Beta | Se | Pval | Sample  size | F |
| --- | --- | --- | --- | --- | --- | --- | --- | --- |
| rs6673880 | G | A | 0.495 | 0.062 | 0.009 | 7.19E-12 | 127,906 | 46.871 |
| rs3795310 | T | C | 0.463 | -0.051 | 0.009 | 5.75E-09 | 127,906 | 34.360 |
| rs56335113 | G | A | 0.697 | -0.065 | 0.009 | 6.02E-12 | 127,906 | 47.377 |
| rs4653164 | T | C | 0.668 | 0.051 | 0.009 | 3.08E-08 | 127,906 | 30.855 |
| rs11210892 | A | G | 0.67 | -0.064 | 0.009 | 2.68E-12 | 127,906 | 48.694 |
| rs1892346 | A | T | 0.564 | 0.048 | 0.009 | 3.56E-08 | 127,906 | 30.253 |
| rs12129573 | A | C | 0.366 | 0.078 | 0.009 | 2.28E-18 | 127,906 | 76.414 |
| rs11165867 | T | C | 0.16 | 0.074 | 0.012 | 1.30E-10 | 127,906 | 41.030 |
| rs1198588 | T | A | 0.788 | 0.103 | 0.011 | 1.73E-21 | 127,906 | 90.246 |
| rs12138231 | A | T | 0.817 | 0.067 | 0.012 | 7.99E-09 | 127,906 | 33.356 |
| rs16851048 | C | T | 0.195 | 0.074 | 0.011 | 4.15E-12 | 127,906 | 48.475 |
| rs7515363 | T | C | 0.619 | -0.054 | 0.009 | 1.84E-09 | 127,906 | 36.139 |
| rs11587347 | G | C | 0.094 | 0.104 | 0.015 | 1.53E-12 | 127,906 | 49.952 |
| rs145071536 | C | T | 0.192 | 0.085 | 0.012 | 1.62E-12 | 127,906 | 50.292 |
| rs6715366 | A | G | 0.267 | 0.054 | 0.010 | 2.49E-08 | 127,906 | 31.103 |
| rs12712510 | C | T | 0.527 | -0.057 | 0.009 | 5.14E-11 | 127,906 | 43.531 |
| rs3770754 | G | C | 0.368 | -0.053 | 0.009 | 5.35E-09 | 127,906 | 33.788 |
| rs13011472 | G | C | 0.484 | 0.070 | 0.009 | 4.28E-16 | 127,906 | 65.482 |
| rs6546857 | G | A | 0.232 | 0.060 | 0.010 | 2.74E-09 | 127,906 | 35.062 |
| rs6721531 | T | A | 0.341 | -0.052 | 0.009 | 1.47E-08 | 127,906 | 32.278 |
| rs7575796 | G | A | 0.094 | -0.096 | 0.017 | 2.06E-08 | 127,906 | 31.347 |
| rs13016542 | C | T | 0.136 | -0.088 | 0.013 | 8.28E-12 | 127,906 | 46.858 |
| rs1881046 | T | G | 0.34 | -0.051 | 0.009 | 3.39E-08 | 127,906 | 30.373 |
| rs2909457 | A | G | 0.564 | -0.049 | 0.009 | 1.48E-08 | 127,906 | 31.721 |
| rs62183855 | C | A | 0.192 | -0.066 | 0.011 | 2.66E-09 | 127,906 | 35.458 |
| rs11693094 | T | C | 0.461 | -0.054 | 0.009 | 4.29E-10 | 127,906 | 39.103 |
| rs12151767 | A | G | 0.487 | -0.061 | 0.009 | 1.31E-12 | 127,906 | 50.484 |
| rs1451488 | G | A | 0.555 | 0.071 | 0.009 | 4.47E-16 | 127,906 | 66.403 |
| rs3739118 | A | G | 0.291 | -0.057 | 0.010 | 2.36E-09 | 127,906 | 36.005 |
| rs3791710 | C | T | 0.203 | -0.060 | 0.011 | 3.02E-08 | 127,906 | 30.868 |
| rs778371 | G | A | 0.286 | 0.081 | 0.010 | 1.49E-17 | 127,906 | 71.987 |
| rs17194490 | T | G | 0.161 | 0.078 | 0.012 | 1.80E-11 | 127,906 | 45.445 |
| rs12489270 | C | T | 0.375 | 0.058 | 0.009 | 7.47E-11 | 127,906 | 42.330 |
| rs1430894 | T | C | 0.483 | 0.053 | 0.009 | 6.15E-10 | 127,906 | 38.404 |
| rs6549963 | C | T | 0.416 | -0.048 | 0.009 | 4.31E-08 | 127,906 | 30.130 |
| rs9876421 | T | C | 0.343 | 0.063 | 0.009 | 9.19E-12 | 127,906 | 46.156 |
| rs2710323 | C | T | 0.49 | -0.078 | 0.009 | 1.23E-19 | 127,906 | 83.116 |
| rs6798742 | G | A | 0.307 | 0.061 | 0.009 | 4.57E-11 | 127,906 | 43.162 |
| rs60135207 | T | G | 0.415 | -0.050 | 0.009 | 1.53E-08 | 127,906 | 31.768 |
| rs167924 | G | A | 0.631 | 0.050 | 0.009 | 2.34E-08 | 127,906 | 31.111 |
| rs1604060 | G | A | 0.885 | 0.077 | 0.014 | 3.24E-08 | 127,906 | 30.411 |
| rs7634476 | G | A | 0.586 | 0.058 | 0.009 | 5.46E-11 | 127,906 | 42.997 |
| rs308697 | A | C | 0.436 | -0.050 | 0.009 | 8.83E-09 | 127,906 | 33.166 |
| rs7647398 | T | C | 0.2 | -0.077 | 0.011 | 1.07E-12 | 127,906 | 50.551 |
| rs35734242 | C | T | 0.426 | 0.051 | 0.009 | 1.37E-08 | 127,906 | 32.457 |
| rs215412 | A | G | 0.328 | 0.058 | 0.009 | 2.69E-10 | 127,906 | 40.209 |
| rs11941714 | A | G | 0.336 | -0.052 | 0.009 | 3.07E-08 | 127,906 | 30.779 |
| rs13107325 | T | C | 0.067 | 0.159 | 0.017 | 2.90E-21 | 127,906 | 89.238 |
| rs1427633 | C | G | 0.593 | -0.048 | 0.009 | 4.10E-08 | 127,906 | 30.130 |
| rs2333321 | G | A | 0.792 | -0.071 | 0.011 | 1.25E-11 | 127,906 | 45.986 |
| rs10035564 | G | A | 0.331 | 0.067 | 0.009 | 4.38E-13 | 127,906 | 52.724 |
| rs4700418 | G | C | 0.493 | 0.070 | 0.009 | 5.37E-16 | 127,906 | 65.103 |
| rs16867571 | G | A | 0.235 | -0.066 | 0.010 | 2.68E-10 | 127,906 | 39.913 |
| rs1901512 | C | T | 0.699 | -0.058 | 0.009 | 5.72E-10 | 127,906 | 38.600 |
| rs187557 | T | C | 0.848 | -0.067 | 0.012 | 2.03E-08 | 127,906 | 31.412 |
| rs10117 | A | G | 0.398 | -0.055 | 0.009 | 4.66E-10 | 127,906 | 39.062 |
| rs9687282 | G | T | 0.334 | 0.053 | 0.009 | 7.33E-09 | 127,906 | 33.410 |
| rs72802868 | T | G | 0.294 | -0.069 | 0.010 | 4.55E-13 | 127,906 | 51.959 |
| rs11740474 | T | A | 0.415 | 0.054 | 0.009 | 1.13E-09 | 127,906 | 37.232 |
| rs12652777 | C | T | 0.522 | -0.049 | 0.009 | 1.52E-08 | 127,906 | 32.199 |
| rs13195636 | C | A | 0.091 | -0.211 | 0.016 | 6.55E-40 | 127,906 | 175.278 |
| rs9461916 | C | T | 0.594 | 0.053 | 0.009 | 1.64E-09 | 127,906 | 36.679 |
| rs2815731 | A | C | 0.358 | -0.060 | 0.009 | 4.39E-11 | 127,906 | 43.478 |
| rs217336 | A | C | 0.434 | -0.050 | 0.009 | 8.05E-09 | 127,906 | 33.431 |
| rs634940 | T | G | 0.252 | 0.066 | 0.010 | 1.78E-11 | 127,906 | 44.980 |
| rs58120505 | C | T | 0.418 | -0.090 | 0.009 | 2.24E-24 | 127,906 | 103.676 |
| rs79210963 | C | T | 0.108 | 0.086 | 0.014 | 4.14E-10 | 127,906 | 39.041 |
| rs1914399 | G | C | 0.521 | -0.049 | 0.009 | 1.40E-08 | 127,906 | 31.857 |
| rs6943762 | C | T | 0.13 | -0.105 | 0.013 | 1.57E-15 | 127,906 | 63.393 |
| rs13233308 | T | C | 0.484 | -0.049 | 0.009 | 1.75E-08 | 127,906 | 32.073 |
| rs2252074 | G | T | 0.395 | 0.069 | 0.009 | 6.19E-15 | 127,906 | 60.599 |
| rs6974218 | C | A | 0.382 | -0.055 | 0.009 | 6.80E-10 | 127,906 | 38.044 |
| rs11534045 | A | G | 0.325 | -0.063 | 0.009 | 1.40E-11 | 127,906 | 45.594 |
| rs1593304 | G | A | 0.801 | 0.064 | 0.011 | 7.45E-09 | 127,906 | 33.349 |
| rs7798283 | G | T | 0.133 | -0.074 | 0.013 | 3.49E-08 | 127,906 | 30.499 |
| rs728055 | A | T | 0.358 | -0.067 | 0.009 | 8.85E-14 | 127,906 | 56.078 |
| rs10503253 | A | C | 0.206 | 0.062 | 0.011 | 4.53E-09 | 127,906 | 34.215 |
| rs4921741 | G | A | 0.261 | 0.056 | 0.010 | 1.21E-08 | 127,906 | 32.652 |
| rs73229090 | A | C | 0.112 | -0.103 | 0.014 | 4.34E-13 | 127,906 | 52.208 |
| rs79445414 | C | T | 0.04 | 0.123 | 0.022 | 2.80E-08 | 127,906 | 30.898 |
| rs6984242 | A | G | 0.6 | -0.055 | 0.009 | 3.85E-10 | 127,906 | 39.526 |
| rs10957321 | A | G | 0.511 | 0.048 | 0.009 | 3.48E-08 | 127,906 | 30.628 |
| rs1915019 | G | A | 0.75 | -0.057 | 0.010 | 6.57E-09 | 127,906 | 33.947 |
| rs10086619 | G | A | 0.161 | 0.072 | 0.012 | 4.97E-10 | 127,906 | 38.745 |
| rs4129585 | C | A | 0.562 | -0.075 | 0.009 | 5.11E-18 | 127,906 | 74.309 |
| rs11136325 | A | G | 0.579 | -0.054 | 0.009 | 3.05E-09 | 127,906 | 34.948 |
| rs498591 | T | A | 0.146 | 0.072 | 0.012 | 2.11E-09 | 127,906 | 35.896 |
| rs505061 | A | C | 0.488 | 0.053 | 0.009 | 5.80E-10 | 127,906 | 38.694 |
| rs2381411 | C | T | 0.402 | 0.050 | 0.009 | 1.25E-08 | 127,906 | 32.800 |
| rs500102 | C | T | 0.591 | -0.052 | 0.009 | 4.87E-09 | 127,906 | 34.516 |
| rs3824451 | C | T | 0.153 | 0.066 | 0.012 | 2.54E-08 | 127,906 | 30.901 |
| rs2078266 | G | A | 0.829 | -0.070 | 0.013 | 2.94E-08 | 127,906 | 30.513 |
| rs17731 | A | G | 0.365 | 0.052 | 0.009 | 4.37E-09 | 127,906 | 34.663 |
| rs6482437 | C | A | 0.888 | 0.099 | 0.014 | 3.33E-12 | 127,906 | 48.512 |
| rs12771371 | A | G | 0.311 | -0.052 | 0.009 | 1.94E-08 | 127,906 | 31.750 |
| rs61857878 | T | A | 0.254 | -0.060 | 0.010 | 4.44E-09 | 127,906 | 34.715 |
| rs11191580 | C | T | 0.09 | -0.132 | 0.016 | 1.77E-17 | 127,906 | 72.198 |
| rs11027839 | C | A | 0.5 | 0.052 | 0.009 | 2.40E-09 | 127,906 | 35.866 |
| rs4636654 | A | G | 0.402 | -0.048 | 0.009 | 4.89E-08 | 127,906 | 29.457 |
| rs12285419 | A | C | 0.188 | 0.085 | 0.011 | 1.05E-14 | 127,906 | 59.577 |
| rs708228 | T | C | 0.329 | 0.053 | 0.009 | 6.56E-09 | 127,906 | 33.665 |
| rs72943392 | C | G | 0.289 | 0.053 | 0.010 | 2.39E-08 | 127,906 | 31.052 |
| rs2514218 | T | C | 0.344 | -0.070 | 0.009 | 1.35E-14 | 127,906 | 58.715 |
| rs12293670 | G | A | 0.34 | -0.070 | 0.009 | 1.56E-14 | 127,906 | 58.715 |
| rs7112616 | C | T | 0.498 | -0.052 | 0.009 | 1.52E-09 | 127,906 | 36.847 |
| rs3802924 | C | A | 0.212 | -0.074 | 0.011 | 9.58E-12 | 127,906 | 46.446 |
| rs11223774 | G | A | 0.706 | -0.052 | 0.009 | 2.74E-08 | 127,906 | 31.191 |
| rs1860002 | T | C | 0.543 | -0.084 | 0.009 | 1.04E-21 | 127,906 | 92.776 |
| rs10876446 | C | G | 0.312 | 0.054 | 0.009 | 1.03E-08 | 127,906 | 33.004 |
| rs61937595 | T | C | 0.092 | -0.130 | 0.016 | 1.15E-15 | 127,906 | 64.493 |
| rs12303743 | C | G | 0.094 | 0.087 | 0.015 | 1.59E-09 | 127,906 | 36.414 |
| rs6538539 | T | G | 0.549 | -0.057 | 0.009 | 4.43E-11 | 127,906 | 43.615 |
| rs10861176 | A | G | 0.737 | 0.056 | 0.010 | 1.59E-08 | 127,906 | 32.075 |
| rs4766428 | T | C | 0.441 | 0.075 | 0.009 | 3.93E-17 | 127,906 | 71.021 |
| rs1615350 | T | C | 0.744 | -0.074 | 0.010 | 4.92E-14 | 127,906 | 56.409 |
| rs12833624 | T | C | 0.342 | 0.050 | 0.009 | 2.77E-08 | 127,906 | 31.111 |
| rs2455415 | T | C | 0.413 | 0.049 | 0.009 | 1.69E-08 | 127,906 | 31.634 |
| rs12877581 | C | G | 0.277 | 0.060 | 0.010 | 1.80E-09 | 127,906 | 36.245 |
| rs9318627 | C | A | 0.4 | -0.061 | 0.009 | 4.35E-12 | 127,906 | 48.363 |
| rs1953205 | A | T | 0.485 | 0.050 | 0.009 | 2.21E-08 | 127,906 | 31.442 |
| rs12883788 | T | C | 0.458 | 0.061 | 0.009 | 1.86E-12 | 127,906 | 49.648 |
| rs2999392 | T | C | 0.691 | 0.052 | 0.009 | 3.05E-08 | 127,906 | 30.366 |
| rs2332700 | G | C | 0.753 | -0.075 | 0.010 | 3.88E-14 | 127,906 | 57.542 |
| rs1540840 | C | G | 0.475 | -0.056 | 0.009 | 2.21E-09 | 127,906 | 35.871 |
| rs10873538 | G | T | 0.334 | 0.067 | 0.009 | 3.01E-13 | 127,906 | 53.407 |
| rs56205728 | A | G | 0.287 | 0.063 | 0.010 | 1.01E-10 | 127,906 | 42.188 |
| rs62018952 | C | T | 0.726 | 0.058 | 0.010 | 1.94E-09 | 127,906 | 36.251 |
| rs35351411 | C | A | 0.544 | 0.064 | 0.009 | 2.21E-13 | 127,906 | 53.281 |
| rs2456020 | T | C | 0.241 | -0.082 | 0.010 | 1.13E-15 | 127,906 | 63.997 |
| rs4779050 | G | T | 0.633 | -0.058 | 0.009 | 7.27E-11 | 127,906 | 42.463 |
| rs4702 | A | G | 0.56 | -0.084 | 0.009 | 2.79E-21 | 127,906 | 89.726 |
| rs8055219 | A | G | 0.229 | 0.067 | 0.010 | 5.69E-11 | 127,906 | 43.355 |
| rs3814883 | T | C | 0.467 | -0.067 | 0.009 | 1.58E-14 | 127,906 | 59.481 |
| rs149165 | G | T | 0.446 | -0.048 | 0.009 | 3.00E-08 | 127,906 | 30.694 |
| rs4575535 | G | A | 0.704 | 0.056 | 0.010 | 5.77E-09 | 127,906 | 33.783 |
| rs73292401 | A | T | 0.192 | 0.068 | 0.011 | 5.48E-10 | 127,906 | 38.468 |
| rs57433322 | G | C | 0.122 | -0.083 | 0.014 | 1.99E-09 | 127,906 | 35.741 |
| rs2696466 | G | A | 0.403 | -0.061 | 0.009 | 2.64E-11 | 127,906 | 44.249 |
| rs9304548 | A | C | 0.748 | -0.057 | 0.010 | 1.59E-08 | 127,906 | 32.151 |
| rs4632195 | T | C | 0.52 | 0.047 | 0.009 | 4.59E-08 | 127,906 | 30.118 |
| rs9636107 | G | A | 0.471 | 0.070 | 0.009 | 5.11E-16 | 127,906 | 66.057 |
| rs11664298 | A | G | 0.193 | 0.077 | 0.011 | 8.94E-13 | 127,906 | 51.360 |
| rs76838079 | T | C | 0.146 | 0.078 | 0.014 | 1.53E-08 | 127,906 | 31.951 |
| rs72986630 | T | C | 0.068 | 0.112 | 0.018 | 3.59E-10 | 127,906 | 39.357 |
| rs1000237 | A | T | 0.355 | 0.073 | 0.009 | 2.80E-16 | 127,906 | 67.656 |
| rs2053079 | G | A | 0.237 | 0.060 | 0.010 | 3.01E-09 | 127,906 | 35.171 |
| rs7251 | G | C | 0.328 | -0.064 | 0.009 | 8.29E-12 | 127,906 | 46.502 |
| rs4812325 | A | G | 0.616 | 0.072 | 0.009 | 8.96E-16 | 127,906 | 65.272 |
| rs11696755 | C | T | 0.184 | 0.064 | 0.011 | 7.26E-09 | 127,906 | 33.531 |
| rs113264400 | C | T | 0.048 | 0.112 | 0.020 | 2.86E-08 | 127,906 | 30.905 |
| rs132582 | T | C | 0.545 | -0.051 | 0.009 | 3.26E-09 | 127,906 | 35.164 |
| rs5751191 | C | T | 0.501 | 0.066 | 0.009 | 3.00E-14 | 127,906 | 58.176 |
| rs8138941 | A | G | 0.204 | 0.058 | 0.011 | 4.46E-08 | 127,906 | 30.038 |
| rs6010045 | C | T | 0.698 | 0.055 | 0.010 | 7.44E-09 | 127,906 | 33.396 |

**Table S4. 270 SNPs associated with hypertension.**

| SNP | Effect  allele | Other allele | Effect allele  frequency | Beta | Se | Pval | Sample  size | F |
| --- | --- | --- | --- | --- | --- | --- | --- | --- |
| rs2493292 | T | C | 0.109 | 0.051 | 0.008 | 3.20E-10 | 500,264 | 39.549 |
| rs4908568 | C | A | 0.525 | -0.028 | 0.005 | 3.56E-08 | 500,264 | 30.374 |
| rs880315 | C | T | 0.413 | 0.072 | 0.005 | 2.43E-45 | 500,264 | 199.696 |
| rs72640287 | T | C | 0.077 | -0.112 | 0.010 | 4.78E-32 | 500,264 | 138.838 |
| rs11249244 | A | G | 0.601 | 0.035 | 0.005 | 1.83E-11 | 500,264 | 45.141 |
| rs12724052 | A | G | 0.134 | 0.047 | 0.007 | 1.33E-10 | 500,264 | 41.269 |
| rs7412540 | C | T | 0.101 | -0.050 | 0.008 | 1.97E-09 | 500,264 | 36.006 |
| rs150711386 | T | C | 0.043 | -0.069 | 0.012 | 3.17E-08 | 500,264 | 30.603 |
| rs2105028 | T | C | 0.379 | 0.030 | 0.005 | 6.09E-09 | 500,264 | 33.805 |
| rs11211570 | C | A | 0.118 | 0.052 | 0.008 | 1.98E-11 | 500,264 | 44.989 |
| rs10888974 | C | T | 0.370 | 0.043 | 0.005 | 2.86E-16 | 500,264 | 66.896 |
| rs2806430 | G | C | 0.795 | 0.034 | 0.006 | 4.65E-08 | 500,264 | 29.857 |
| rs10922477 | A | C | 0.670 | -0.033 | 0.005 | 7.51E-10 | 500,264 | 37.884 |
| rs509705 | G | A | 0.288 | 0.031 | 0.006 | 3.77E-08 | 500,264 | 30.262 |
| rs10776752 | T | G | 0.171 | 0.110 | 0.007 | 4.53E-61 | 500,264 | 271.831 |
| rs7552539 | G | A | 0.119 | 0.047 | 0.008 | 1.42E-09 | 500,264 | 36.635 |
| rs77267109 | C | A | 0.015 | 0.142 | 0.021 | 5.25E-12 | 500,264 | 47.592 |
| rs72702577 | C | T | 0.098 | -0.050 | 0.008 | 4.26E-09 | 500,264 | 34.502 |
| rs74593044 | G | C | 0.079 | 0.056 | 0.009 | 1.57E-09 | 500,264 | 36.443 |
| rs10912903 | G | C | 0.575 | -0.033 | 0.005 | 1.46E-10 | 500,264 | 41.076 |
| rs1745414 | A | T | 0.566 | 0.038 | 0.005 | 4.70E-14 | 500,264 | 56.851 |
| rs1317181 | T | G | 0.210 | 0.060 | 0.006 | 1.25E-22 | 500,264 | 95.829 |
| rs34004783 | G | C | 0.594 | -0.081 | 0.005 | 5.40E-56 | 500,264 | 248.541 |
| rs35784342 | T | C | 0.579 | 0.036 | 0.005 | 2.55E-12 | 500,264 | 49.007 |
| rs3738867 | C | T | 0.026 | 0.091 | 0.016 | 7.06E-09 | 500,264 | 33.517 |
| rs2888854 | A | G | 0.682 | -0.040 | 0.005 | 1.47E-13 | 500,264 | 54.612 |
| rs513386 | T | A | 0.213 | 0.039 | 0.006 | 3.94E-10 | 500,264 | 39.141 |
| rs4672324 | T | A | 0.518 | -0.032 | 0.005 | 2.19E-10 | 500,264 | 40.287 |
| rs115327327 | T | A | 0.381 | -0.033 | 0.005 | 1.31E-10 | 500,264 | 41.293 |
| rs17174870 | T | C | 0.376 | -0.031 | 0.005 | 1.87E-09 | 500,264 | 36.102 |
| rs2119149 | C | T | 0.118 | -0.045 | 0.008 | 1.14E-08 | 500,264 | 32.591 |
| rs55944332 | G | A | 0.257 | 0.040 | 0.006 | 2.86E-12 | 500,264 | 48.783 |
| rs3771612 | A | G | 0.866 | -0.042 | 0.007 | 1.61E-08 | 500,264 | 31.917 |
| rs116099394 | T | G | 0.146 | -0.059 | 0.007 | 1.24E-16 | 500,264 | 68.546 |
| rs2704368 | G | A | 0.837 | -0.064 | 0.007 | 4.43E-21 | 500,264 | 88.772 |
| rs268263 | A | T | 0.794 | 0.045 | 0.006 | 2.84E-13 | 500,264 | 53.316 |
| rs16867335 | T | C | 0.280 | 0.033 | 0.006 | 2.13E-09 | 500,264 | 35.849 |
| rs4362519 | C | G | 0.362 | -0.034 | 0.005 | 1.46E-10 | 500,264 | 41.084 |
| rs16828714 | T | G | 0.358 | -0.029 | 0.005 | 3.27E-08 | 500,264 | 30.538 |
| rs116247686 | G | T | 0.018 | -0.110 | 0.019 | 1.39E-08 | 500,264 | 32.200 |
| rs78058190 | A | G | 0.083 | 0.065 | 0.009 | 1.38E-12 | 500,264 | 50.209 |
| rs1515114 | G | A | 0.429 | 0.038 | 0.005 | 1.31E-13 | 500,264 | 54.841 |
| rs1000010 | G | A | 0.410 | -0.034 | 0.005 | 4.85E-11 | 500,264 | 43.238 |
| rs4685218 | T | C | 0.099 | 0.060 | 0.008 | 1.03E-12 | 500,264 | 50.783 |
| rs9860251 | T | A | 0.083 | 0.053 | 0.009 | 5.39E-09 | 500,264 | 34.045 |
| rs2643826 | T | C | 0.404 | 0.050 | 0.005 | 1.42E-22 | 500,264 | 95.583 |
| rs58783993 | C | A | 0.324 | 0.031 | 0.005 | 8.15E-09 | 500,264 | 33.239 |
| rs1996097 | C | T | 0.379 | -0.034 | 0.005 | 6.86E-11 | 500,264 | 42.559 |
| rs3821843 | A | G | 0.659 | 0.035 | 0.005 | 4.07E-11 | 500,264 | 43.578 |
| rs9836592 | T | C | 0.651 | 0.034 | 0.005 | 1.41E-10 | 500,264 | 41.153 |
| rs73226528 | C | T | 0.282 | 0.034 | 0.006 | 1.57E-09 | 500,264 | 36.440 |
| rs9851392 | A | T | 0.551 | 0.036 | 0.005 | 1.44E-12 | 500,264 | 50.132 |
| rs6795376 | T | C | 0.445 | 0.031 | 0.005 | 7.63E-10 | 500,264 | 37.852 |
| rs6808266 | G | A | 0.799 | 0.046 | 0.006 | 1.78E-13 | 500,264 | 54.233 |
| rs11715377 | T | A | 0.079 | -0.059 | 0.009 | 2.46E-10 | 500,264 | 40.060 |
| rs10804833 | A | G | 0.497 | -0.032 | 0.005 | 3.49E-10 | 500,264 | 39.380 |
| rs73249866 | G | A | 0.260 | -0.037 | 0.006 | 8.49E-11 | 500,264 | 42.141 |
| rs2291434 | T | G | 0.439 | -0.031 | 0.005 | 7.47E-10 | 500,264 | 37.894 |
| rs55893990 | G | A | 0.318 | 0.033 | 0.005 | 1.47E-09 | 500,264 | 36.571 |
| rs12509595 | C | T | 0.314 | 0.092 | 0.005 | 8.31E-64 | 500,264 | 284.386 |
| rs17010957 | C | T | 0.152 | 0.052 | 0.007 | 1.39E-13 | 500,264 | 54.717 |
| rs4426786 | G | A | 0.953 | -0.069 | 0.012 | 5.66E-09 | 500,264 | 33.948 |
| rs13112725 | C | G | 0.829 | 0.056 | 0.007 | 8.51E-17 | 500,264 | 69.287 |
| rs12503104 | T | C | 0.446 | -0.029 | 0.005 | 1.60E-08 | 500,264 | 31.933 |
| rs1879053 | C | T | 0.548 | -0.042 | 0.005 | 1.02E-16 | 500,264 | 68.930 |
| rs1836039 | G | A | 0.149 | -0.046 | 0.007 | 5.29E-11 | 500,264 | 43.068 |
| rs28695597 | A | C | 0.452 | 0.028 | 0.005 | 1.95E-08 | 500,264 | 31.543 |
| rs4690777 | C | T | 0.643 | -0.039 | 0.005 | 1.49E-13 | 500,264 | 54.581 |
| rs6537481 | G | A | 0.186 | 0.039 | 0.006 | 1.11E-09 | 500,264 | 37.128 |
| rs4691670 | T | C | 0.399 | -0.035 | 0.005 | 8.19E-12 | 500,264 | 46.720 |
| rs72689147 | T | G | 0.218 | -0.069 | 0.006 | 8.20E-30 | 500,264 | 128.622 |
| rs60776549 | G | A | 0.275 | 0.037 | 0.006 | 3.62E-11 | 500,264 | 43.809 |
| rs12656497 | C | T | 0.586 | 0.059 | 0.005 | 1.09E-30 | 500,264 | 132.627 |
| rs188155432 | T | C | 0.024 | 0.154 | 0.016 | 8.26E-21 | 500,264 | 87.538 |
| rs3857275 | C | T | 0.256 | -0.036 | 0.006 | 6.51E-10 | 500,264 | 38.163 |
| rs10050467 | T | C | 0.408 | -0.029 | 0.005 | 1.42E-08 | 500,264 | 32.163 |
| rs1819074 | A | G | 0.666 | -0.033 | 0.005 | 4.04E-10 | 500,264 | 39.094 |
| rs2549805 | A | T | 0.808 | 0.056 | 0.006 | 3.31E-18 | 500,264 | 75.693 |
| rs7732228 | G | C | 0.422 | -0.030 | 0.005 | 4.38E-09 | 500,264 | 34.446 |
| rs10077410 | A | G | 0.227 | 0.046 | 0.006 | 1.75E-14 | 500,264 | 58.799 |
| rs6860901 | T | C | 0.298 | 0.059 | 0.005 | 9.41E-27 | 500,264 | 114.646 |
| rs982085 | G | C | 0.549 | 0.028 | 0.005 | 1.85E-08 | 500,264 | 31.643 |
| rs9763488 | T | C | 0.386 | 0.033 | 0.005 | 1.06E-10 | 500,264 | 41.705 |
| rs113700836 | T | C | 0.195 | 0.043 | 0.006 | 1.59E-11 | 500,264 | 45.419 |
| rs4371736 | C | G | 0.382 | -0.047 | 0.005 | 4.63E-20 | 500,264 | 84.132 |
| rs4565196 | A | G | 0.121 | 0.043 | 0.008 | 2.89E-08 | 500,264 | 30.777 |
| rs1345721 | C | G | 0.185 | -0.039 | 0.006 | 1.51E-09 | 500,264 | 36.526 |
| rs11134677 | G | A | 0.665 | 0.033 | 0.005 | 9.02E-10 | 500,264 | 37.526 |
| rs10073078 | A | G | 0.535 | -0.028 | 0.005 | 4.75E-08 | 500,264 | 29.818 |
| rs2569882 | C | T | 0.376 | -0.033 | 0.005 | 2.73E-10 | 500,264 | 39.857 |
| rs9396569 | A | G | 0.158 | 0.038 | 0.007 | 3.79E-08 | 500,264 | 30.255 |
| rs2791973 | C | T | 0.628 | -0.030 | 0.005 | 5.65E-09 | 500,264 | 33.953 |
| rs111663960 | G | A | 0.109 | 0.062 | 0.008 | 1.43E-14 | 500,264 | 59.187 |
| rs2596472 | A | G | 0.682 | 0.047 | 0.005 | 6.09E-18 | 500,264 | 74.491 |
| rs6901866 | C | T | 0.284 | 0.048 | 0.006 | 4.46E-18 | 500,264 | 75.108 |
| rs1358980 | T | C | 0.463 | 0.038 | 0.005 | 5.94E-14 | 500,264 | 56.392 |
| rs6927317 | C | T | 0.399 | 0.035 | 0.005 | 1.63E-11 | 500,264 | 45.372 |
| rs6915002 | C | T | 0.629 | -0.029 | 0.005 | 3.32E-08 | 500,264 | 30.509 |
| rs536014705 | T | G | 0.038 | 0.072 | 0.013 | 3.64E-08 | 500,264 | 30.334 |
| rs1413969 | G | A | 0.411 | 0.043 | 0.005 | 3.51E-17 | 500,264 | 71.036 |
| rs6918791 | G | C | 0.728 | 0.044 | 0.006 | 1.40E-14 | 500,264 | 59.227 |
| rs62426324 | T | C | 0.501 | 0.061 | 0.005 | 2.61E-33 | 500,264 | 144.616 |
| rs198645 | T | A | 0.332 | -0.038 | 0.005 | 1.49E-12 | 500,264 | 50.058 |
| rs17080102 | C | G | 0.076 | -0.096 | 0.010 | 3.82E-24 | 500,264 | 102.739 |
| rs73030239 | G | A | 0.056 | -0.073 | 0.011 | 4.81E-11 | 500,264 | 43.252 |
| rs73032446 | T | C | 0.092 | -0.049 | 0.009 | 2.56E-08 | 500,264 | 31.017 |
| rs57234495 | A | G | 0.250 | -0.037 | 0.006 | 1.73E-10 | 500,264 | 40.755 |
| rs4724960 | G | A | 0.440 | 0.036 | 0.005 | 1.43E-12 | 500,264 | 50.137 |
| rs3757666 | G | C | 0.325 | 0.034 | 0.005 | 2.56E-10 | 500,264 | 39.985 |
| rs2699803 | C | T | 0.535 | 0.037 | 0.005 | 1.65E-13 | 500,264 | 54.386 |
| rs3735533 | C | T | 0.905 | 0.127 | 0.009 | 2.14E-48 | 500,264 | 213.702 |
| rs11563582 | A | G | 0.147 | 0.071 | 0.007 | 1.94E-23 | 500,264 | 99.526 |
| rs28361576 | A | G | 0.035 | 0.080 | 0.014 | 5.24E-09 | 500,264 | 34.099 |
| rs62455791 | T | A | 0.188 | -0.040 | 0.006 | 5.21E-10 | 500,264 | 38.596 |
| rs36036189 | C | T | 0.803 | -0.038 | 0.006 | 1.40E-09 | 500,264 | 36.672 |
| rs17477177 | C | T | 0.298 | 0.051 | 0.005 | 8.33E-21 | 500,264 | 87.524 |
| rs193686 | T | C | 0.719 | -0.038 | 0.006 | 5.97E-12 | 500,264 | 47.340 |
| rs56179563 | A | G | 0.354 | -0.032 | 0.005 | 1.20E-09 | 500,264 | 36.976 |
| rs75511781 | G | A | 0.079 | 0.068 | 0.009 | 4.81E-13 | 500,264 | 52.283 |
| rs57274670 | C | G | 0.265 | -0.038 | 0.006 | 3.99E-11 | 500,264 | 43.619 |
| rs3918226 | T | C | 0.070 | 0.160 | 0.010 | 1.76E-60 | 500,264 | 269.122 |
| rs10253736 | T | C | 0.231 | 0.045 | 0.006 | 2.89E-14 | 500,264 | 57.811 |
| rs62494194 | A | G | 0.450 | 0.030 | 0.005 | 2.66E-09 | 500,264 | 35.418 |
| rs2442466 | G | C | 0.505 | 0.030 | 0.005 | 3.24E-09 | 500,264 | 35.031 |
| rs7831557 | A | G | 0.466 | -0.044 | 0.005 | 2.11E-18 | 500,264 | 76.580 |
| rs10086575 | A | G | 0.296 | -0.039 | 0.006 | 1.84E-12 | 500,264 | 49.645 |
| rs2725366 | A | G | 0.620 | -0.030 | 0.005 | 7.42E-09 | 500,264 | 33.421 |
| rs17310011 | G | C | 0.545 | 0.028 | 0.005 | 4.63E-08 | 500,264 | 29.867 |
| rs2977324 | G | T | 0.718 | 0.033 | 0.006 | 2.77E-09 | 500,264 | 35.343 |
| rs6986186 | G | A | 0.138 | 0.040 | 0.007 | 3.77E-08 | 500,264 | 30.265 |
| rs2515236 | G | A | 0.391 | 0.037 | 0.005 | 9.32E-13 | 500,264 | 50.982 |
| rs35783704 | A | G | 0.155 | -0.064 | 0.007 | 3.59E-20 | 500,264 | 84.635 |
| rs11785859 | G | A | 0.266 | -0.033 | 0.006 | 6.92E-09 | 500,264 | 33.557 |
| rs3802228 | G | A | 0.569 | -0.035 | 0.005 | 4.07E-12 | 500,264 | 48.088 |
| rs628919 | A | C | 0.540 | -0.030 | 0.005 | 4.74E-09 | 500,264 | 34.292 |
| rs1330298 | G | A | 0.213 | 0.034 | 0.006 | 2.07E-08 | 500,264 | 31.427 |
| rs1412834 | C | T | 0.434 | 0.039 | 0.005 | 1.43E-14 | 500,264 | 59.187 |
| rs7874497 | G | A | 0.628 | 0.043 | 0.005 | 3.67E-16 | 500,264 | 66.404 |
| rs67227401 | T | C | 0.100 | -0.060 | 0.008 | 1.05E-12 | 500,264 | 50.742 |
| rs10217559 | T | C | 0.694 | 0.037 | 0.005 | 1.17E-11 | 500,264 | 46.020 |
| rs12375937 | C | G | 0.089 | 0.056 | 0.009 | 3.04E-10 | 500,264 | 39.649 |
| rs9696459 | T | C | 0.319 | -0.031 | 0.006 | 1.14E-08 | 500,264 | 32.580 |
| rs72779268 | A | C | 0.064 | -0.115 | 0.010 | 8.22E-29 | 500,264 | 124.048 |
| rs10739995 | G | A | 0.121 | 0.047 | 0.008 | 1.20E-09 | 500,264 | 36.969 |
| rs57994832 | G | C | 0.242 | -0.038 | 0.006 | 2.26E-10 | 500,264 | 40.229 |
| rs1888693 | A | G | 0.386 | 0.039 | 0.005 | 6.65E-14 | 500,264 | 56.168 |
| rs7070847 | A | G | 0.235 | -0.036 | 0.006 | 1.99E-09 | 500,264 | 35.988 |
| rs188460025 | T | C | 0.070 | -0.065 | 0.010 | 4.05E-11 | 500,264 | 43.592 |
| rs57541197 | A | G | 0.102 | -0.068 | 0.008 | 5.11E-16 | 500,264 | 65.753 |
| rs1848797 | G | A | 0.358 | -0.033 | 0.005 | 5.11E-10 | 500,264 | 38.637 |
| rs35506078 | C | T | 0.350 | 0.030 | 0.005 | 1.95E-08 | 500,264 | 31.544 |
| rs3740051 | G | A | 0.130 | 0.043 | 0.007 | 8.76E-09 | 500,264 | 33.099 |
| rs7088877 | G | C | 0.566 | -0.030 | 0.005 | 1.72E-09 | 500,264 | 36.268 |
| rs2274224 | C | G | 0.344 | -0.046 | 0.005 | 3.91E-18 | 500,264 | 75.365 |
| rs10786736 | C | G | 0.091 | -0.094 | 0.009 | 5.52E-27 | 500,264 | 115.702 |
| rs7898224 | A | G | 0.384 | 0.037 | 0.005 | 1.89E-12 | 500,264 | 49.591 |
| rs1265002 | T | A | 0.952 | -0.095 | 0.012 | 1.02E-15 | 500,264 | 64.390 |
| rs12771120 | C | A | 0.228 | -0.039 | 0.006 | 5.77E-11 | 500,264 | 42.898 |
| rs74157557 | T | A | 0.036 | 0.077 | 0.013 | 1.14E-08 | 500,264 | 32.592 |
| rs2782980 | C | T | 0.654 | 0.060 | 0.005 | 2.14E-29 | 500,264 | 126.722 |
| rs11015994 | T | C | 0.247 | 0.033 | 0.006 | 1.30E-08 | 500,264 | 32.332 |
| rs612652 | C | T | 0.574 | 0.059 | 0.005 | 4.15E-31 | 500,264 | 134.544 |
| rs55640925 | A | C | 0.175 | -0.050 | 0.007 | 8.92E-14 | 500,264 | 55.592 |
| rs72851658 | A | T | 0.197 | 0.052 | 0.006 | 1.41E-16 | 500,264 | 68.292 |
| rs35271178 | T | C | 0.499 | -0.029 | 0.005 | 8.20E-09 | 500,264 | 33.226 |
| rs11029864 | T | C | 0.497 | -0.030 | 0.005 | 3.03E-09 | 500,264 | 35.163 |
| rs4351777 | C | T | 0.848 | -0.045 | 0.007 | 9.89E-11 | 500,264 | 41.843 |
| rs141878040 | T | G | 0.405 | 0.040 | 0.005 | 3.93E-15 | 500,264 | 61.734 |
| rs117232803 | A | G | 0.048 | 0.065 | 0.012 | 3.43E-08 | 500,264 | 30.447 |
| rs7126322 | A | G | 0.309 | 0.030 | 0.005 | 2.09E-08 | 500,264 | 31.405 |
| rs112968778 | T | G | 0.040 | 0.071 | 0.013 | 4.31E-08 | 500,264 | 30.006 |
| rs2868996 | T | A | 0.238 | 0.042 | 0.006 | 1.94E-12 | 500,264 | 49.548 |
| rs7106223 | A | G | 0.191 | -0.042 | 0.006 | 8.82E-11 | 500,264 | 42.066 |
| rs2583477 | T | C | 0.629 | 0.030 | 0.005 | 1.60E-08 | 500,264 | 31.934 |
| rs751984 | C | T | 0.171 | -0.069 | 0.007 | 1.87E-24 | 500,264 | 104.151 |
| rs489574 | A | G | 0.282 | -0.037 | 0.006 | 3.50E-11 | 500,264 | 43.875 |
| rs604723 | C | T | 0.741 | 0.067 | 0.006 | 4.83E-31 | 500,264 | 134.247 |
| rs483657 | T | C | 0.165 | -0.040 | 0.007 | 2.70E-09 | 500,264 | 35.388 |
| rs7123454 | A | C | 0.824 | -0.043 | 0.007 | 9.34E-11 | 500,264 | 41.956 |
| rs665759 | A | G | 0.251 | 0.035 | 0.006 | 1.34E-09 | 500,264 | 36.756 |
| rs7129204 | C | G | 0.151 | 0.048 | 0.007 | 1.09E-11 | 500,264 | 46.157 |
| rs12828438 | G | A | 0.535 | -0.041 | 0.005 | 8.65E-16 | 500,264 | 64.716 |
| rs10846167 | T | C | 0.241 | 0.036 | 0.006 | 7.94E-10 | 500,264 | 37.774 |
| rs114850651 | A | G | 0.039 | -0.094 | 0.013 | 7.50E-13 | 500,264 | 51.408 |
| rs73073676 | T | A | 0.220 | -0.047 | 0.006 | 7.90E-15 | 500,264 | 60.360 |
| rs297935 | G | A | 0.763 | 0.034 | 0.006 | 1.38E-08 | 500,264 | 32.211 |
| rs7967954 | A | G | 0.589 | -0.049 | 0.005 | 1.13E-21 | 500,264 | 91.482 |
| rs78204022 | T | C | 0.053 | 0.072 | 0.011 | 1.66E-10 | 500,264 | 40.833 |
| rs1048565 | T | C | 0.075 | -0.065 | 0.010 | 1.50E-11 | 500,264 | 45.535 |
| rs7139122 | A | G | 0.020 | 0.197 | 0.018 | 1.69E-28 | 500,264 | 122.615 |
| rs754133 | A | G | 0.407 | -0.061 | 0.005 | 1.06E-32 | 500,264 | 141.820 |
| rs941208 | T | C | 0.676 | -0.030 | 0.005 | 2.13E-08 | 500,264 | 31.376 |
| rs112285172 | A | C | 0.199 | -0.036 | 0.006 | 7.48E-09 | 500,264 | 33.405 |
| rs117703053 | A | G | 0.093 | 0.049 | 0.009 | 1.27E-08 | 500,264 | 32.375 |
| rs11105337 | T | A | 0.077 | -0.096 | 0.009 | 3.37E-24 | 500,264 | 102.988 |
| rs6538214 | C | A | 0.507 | -0.028 | 0.005 | 2.77E-08 | 500,264 | 30.861 |
| rs653178 | T | C | 0.586 | -0.067 | 0.005 | 1.10E-39 | 500,264 | 173.791 |
| rs35429 | G | A | 0.365 | -0.047 | 0.005 | 3.07E-19 | 500,264 | 80.395 |
| rs6490021 | C | A | 0.696 | 0.047 | 0.005 | 1.09E-17 | 500,264 | 73.351 |
| rs628137 | C | A | 0.580 | 0.041 | 0.005 | 1.36E-15 | 500,264 | 63.825 |
| rs9508490 | T | C | 0.642 | -0.038 | 0.005 | 4.19E-13 | 500,264 | 52.553 |
| rs9318030 | G | C | 0.098 | 0.048 | 0.008 | 1.10E-08 | 500,264 | 32.648 |
| rs9542771 | G | A | 0.673 | -0.030 | 0.005 | 3.05E-08 | 500,264 | 30.675 |
| rs9590507 | C | T | 0.212 | 0.039 | 0.006 | 1.79E-10 | 500,264 | 40.684 |
| rs8013475 | G | A | 0.167 | 0.037 | 0.007 | 4.29E-08 | 500,264 | 30.013 |
| rs1136165 | T | G | 0.680 | 0.036 | 0.005 | 2.84E-11 | 500,264 | 44.286 |
| rs79622589 | T | C | 0.221 | -0.038 | 0.006 | 4.72E-10 | 500,264 | 38.791 |
| rs3101436 | G | A | 0.650 | -0.041 | 0.005 | 4.42E-15 | 500,264 | 61.503 |
| rs12911761 | T | C | 0.398 | 0.030 | 0.005 | 3.38E-09 | 500,264 | 34.953 |
| rs1984442 | C | T | 0.644 | 0.038 | 0.005 | 2.84E-13 | 500,264 | 53.314 |
| rs117539635 | G | A | 0.017 | -0.120 | 0.020 | 1.88E-09 | 500,264 | 36.098 |
| rs512943 | T | C | 0.144 | -0.043 | 0.007 | 2.55E-09 | 500,264 | 35.503 |
| rs7174038 | G | C | 0.406 | 0.046 | 0.005 | 3.56E-19 | 500,264 | 80.099 |
| rs17807723 | A | G | 0.190 | -0.040 | 0.006 | 5.08E-10 | 500,264 | 38.647 |
| rs6227 | T | C | 0.268 | 0.069 | 0.006 | 3.18E-34 | 500,264 | 148.794 |
| rs74038450 | T | C | 0.068 | 0.055 | 0.010 | 4.31E-08 | 500,264 | 30.007 |
| rs35198836 | T | C | 0.322 | 0.038 | 0.005 | 2.79E-12 | 500,264 | 48.830 |
| rs117645982 | G | C | 0.023 | 0.108 | 0.017 | 1.37E-10 | 500,264 | 41.204 |
| rs8056889 | T | C | 0.441 | -0.029 | 0.005 | 1.83E-08 | 500,264 | 31.662 |
| rs77924615 | A | G | 0.223 | -0.036 | 0.006 | 2.62E-09 | 500,264 | 35.448 |
| rs35490232 | T | C | 0.453 | 0.030 | 0.005 | 3.74E-09 | 500,264 | 34.757 |
| rs1558901 | T | A | 0.428 | 0.062 | 0.005 | 1.59E-34 | 500,264 | 150.176 |
| rs45474499 | T | C | 0.063 | 0.066 | 0.010 | 1.56E-10 | 500,264 | 40.955 |
| rs3975148 | C | A | 0.556 | 0.031 | 0.005 | 1.32E-09 | 500,264 | 36.778 |
| rs2966083 | T | G | 0.587 | -0.031 | 0.005 | 1.86E-09 | 500,264 | 36.114 |
| rs8045875 | C | T | 0.594 | 0.039 | 0.005 | 4.77E-14 | 500,264 | 56.822 |
| rs11645355 | A | G | 0.293 | 0.038 | 0.006 | 4.46E-12 | 500,264 | 47.912 |
| rs222852 | G | A | 0.395 | -0.046 | 0.005 | 6.76E-19 | 500,264 | 78.832 |
| rs2302661 | C | G | 0.488 | -0.036 | 0.005 | 1.39E-12 | 500,264 | 50.191 |
| rs8074938 | G | A | 0.672 | -0.033 | 0.005 | 8.54E-10 | 500,264 | 37.632 |
| rs11874 | A | G | 0.169 | 0.063 | 0.007 | 2.26E-21 | 500,264 | 90.107 |
| rs11650158 | A | G | 0.207 | -0.038 | 0.006 | 6.38E-10 | 500,264 | 38.201 |
| rs9897429 | A | G | 0.517 | 0.033 | 0.005 | 6.50E-11 | 500,264 | 42.664 |
| rs2257205 | T | C | 0.142 | -0.045 | 0.007 | 6.31E-10 | 500,264 | 38.224 |
| rs79538876 | C | T | 0.035 | -0.080 | 0.014 | 5.70E-09 | 500,264 | 33.934 |
| rs2286526 | T | C | 0.713 | 0.049 | 0.006 | 6.98E-19 | 500,264 | 78.770 |
| rs4298 | T | C | 0.092 | -0.066 | 0.009 | 4.71E-14 | 500,264 | 56.849 |
| rs1436138 | G | A | 0.356 | -0.038 | 0.005 | 3.33E-13 | 500,264 | 53.006 |
| rs4296334 | T | C | 0.647 | -0.033 | 0.005 | 5.58E-10 | 500,264 | 38.464 |
| rs66794444 | T | C | 0.109 | 0.053 | 0.008 | 4.52E-11 | 500,264 | 43.377 |
| rs12962116 | G | T | 0.191 | -0.035 | 0.006 | 4.58E-08 | 500,264 | 29.888 |
| rs4599004 | T | C | 0.286 | -0.039 | 0.006 | 1.67E-12 | 500,264 | 49.838 |
| rs1569372 | G | A | 0.490 | 0.033 | 0.005 | 8.63E-11 | 500,264 | 42.109 |
| rs143466522 | A | G | 0.015 | 0.168 | 0.021 | 7.85E-16 | 500,264 | 64.908 |
| rs167479 | G | T | 0.577 | 0.077 | 0.005 | 9.58E-52 | 500,264 | 229.056 |
| rs11667341 | T | C | 0.033 | -0.101 | 0.014 | 1.81E-12 | 500,264 | 49.679 |
| rs13346655 | G | C | 0.217 | 0.044 | 0.006 | 6.49E-13 | 500,264 | 51.694 |
| rs2005873 | T | C | 0.552 | -0.030 | 0.005 | 1.92E-09 | 500,264 | 36.054 |
| rs376510895 | A | G | 0.022 | -0.104 | 0.017 | 1.23E-09 | 500,264 | 36.928 |
| rs76100548 | G | C | 0.637 | 0.029 | 0.005 | 1.81E-08 | 500,264 | 31.686 |
| rs6108165 | A | G | 0.209 | -0.036 | 0.006 | 7.30E-09 | 500,264 | 33.453 |
| rs6040076 | C | G | 0.433 | -0.032 | 0.005 | 2.67E-10 | 500,264 | 39.902 |
| rs62185661 | A | G | 0.242 | 0.053 | 0.006 | 2.28E-19 | 500,264 | 80.979 |
| rs6047267 | A | G | 0.751 | -0.032 | 0.006 | 3.08E-08 | 500,264 | 30.655 |
| rs12481717 | A | G | 0.194 | 0.046 | 0.006 | 5.14E-13 | 500,264 | 52.149 |
| rs6029750 | A | G | 0.376 | -0.029 | 0.005 | 1.89E-08 | 500,264 | 31.606 |
| rs6017285 | G | C | 0.126 | 0.049 | 0.008 | 1.02E-10 | 500,264 | 41.780 |
| rs55850843 | T | C | 0.055 | -0.069 | 0.011 | 6.30E-10 | 500,264 | 38.226 |
| rs117234386 | A | C | 0.074 | 0.057 | 0.010 | 3.41E-09 | 500,264 | 34.934 |
| rs6015445 | C | G | 0.162 | 0.119 | 0.007 | 2.87E-69 | 500,264 | 309.458 |
| rs9679855 | G | A | 0.034 | 0.116 | 0.014 | 7.28E-17 | 500,264 | 69.595 |
| rs180957609 | C | A | 0.034 | -0.088 | 0.014 | 3.24E-10 | 500,264 | 39.527 |
| rs1923031 | C | T | 0.613 | -0.038 | 0.005 | 3.22E-13 | 500,264 | 53.071 |
| rs2823140 | A | G | 0.385 | 0.032 | 0.005 | 4.95E-10 | 500,264 | 38.697 |
| rs2300373 | T | A | 0.185 | 0.038 | 0.006 | 5.37E-09 | 500,264 | 34.049 |
| rs607398 | C | A | 0.291 | 0.037 | 0.006 | 4.15E-11 | 500,264 | 43.541 |
| rs1454651 | G | A | 0.686 | 0.034 | 0.005 | 2.82E-10 | 500,264 | 39.798 |
| rs5752822 | G | A | 0.213 | 0.051 | 0.006 | 1.27E-16 | 500,264 | 68.490 |
| rs78660772 | T | A | 0.172 | 0.039 | 0.007 | 4.25E-09 | 500,264 | 34.507 |
| rs5994376 | C | A | 0.539 | 0.028 | 0.005 | 4.46E-08 | 500,264 | 29.937 |

**Table S5. 232 SNPs associated with essential hypertension.**

| SNP | Effect  allele | Other allele | Effect allele  frequency | Beta | Se | Pval | Sample  size | F |
| --- | --- | --- | --- | --- | --- | --- | --- | --- |
| rs2493292 | T | C | 0.109 | 0.048 | 0.008 | 1.86E-08 | 478,149 | 31.631 |
| rs880315 | C | T | 0.412 | 0.070 | 0.005 | 1.47E-38 | 478,149 | 168.637 |
| rs72640287 | T | C | 0.078 | -0.113 | 0.010 | 3.02E-29 | 478,149 | 126.039 |
| rs6668768 | T | C | 0.215 | 0.035 | 0.006 | 3.98E-08 | 478,149 | 30.159 |
| rs12729652 | A | G | 0.155 | 0.048 | 0.007 | 6.97E-11 | 478,149 | 42.528 |
| rs2105028 | T | C | 0.379 | 0.032 | 0.005 | 5.27E-09 | 478,149 | 34.086 |
| rs11211570 | C | A | 0.117 | 0.056 | 0.008 | 1.49E-11 | 478,149 | 45.542 |
| rs4638151 | T | C | 0.628 | -0.044 | 0.005 | 7.09E-16 | 478,149 | 65.108 |
| rs10922477 | A | C | 0.670 | -0.035 | 0.006 | 4.63E-10 | 478,149 | 38.828 |
| rs10776752 | T | G | 0.170 | 0.108 | 0.007 | 7.66E-54 | 478,149 | 238.672 |
| rs7552539 | G | A | 0.119 | 0.047 | 0.008 | 8.81E-09 | 478,149 | 33.088 |
| rs77267109 | C | A | 0.015 | 0.143 | 0.022 | 3.87E-11 | 478,149 | 43.680 |
| rs72702577 | C | T | 0.098 | -0.050 | 0.009 | 2.36E-08 | 478,149 | 31.175 |
| rs10912903 | G | C | 0.575 | -0.033 | 0.005 | 4.88E-10 | 478,149 | 38.724 |
| rs1745414 | A | T | 0.566 | 0.036 | 0.005 | 1.30E-11 | 478,149 | 45.809 |
| rs1317181 | T | G | 0.209 | 0.061 | 0.006 | 9.78E-21 | 478,149 | 87.205 |
| rs34004783 | G | C | 0.594 | -0.083 | 0.005 | 1.35E-53 | 478,149 | 237.545 |
| rs35784342 | T | C | 0.578 | 0.035 | 0.005 | 7.21E-11 | 478,149 | 42.461 |
| rs3738867 | C | T | 0.026 | 0.097 | 0.017 | 4.47E-09 | 478,149 | 34.409 |
| rs630901 | A | T | 0.304 | 0.034 | 0.006 | 4.31E-09 | 478,149 | 34.480 |
| rs2888854 | A | G | 0.682 | -0.040 | 0.006 | 1.88E-12 | 478,149 | 49.603 |
| rs2589071 | T | A | 0.063 | -0.067 | 0.011 | 1.15E-09 | 478,149 | 37.050 |
| rs4672324 | T | A | 0.518 | -0.031 | 0.005 | 2.97E-09 | 478,149 | 35.203 |
| rs115327327 | T | A | 0.381 | -0.033 | 0.005 | 1.04E-09 | 478,149 | 37.241 |
| rs17174870 | T | C | 0.376 | -0.031 | 0.005 | 2.18E-08 | 478,149 | 31.323 |
| rs34075829 | A | G | 0.153 | -0.041 | 0.007 | 3.76E-08 | 478,149 | 30.269 |
| rs55944332 | G | A | 0.256 | 0.036 | 0.006 | 2.03E-09 | 478,149 | 35.949 |
| rs270952 | G | T | 0.792 | 0.036 | 0.007 | 3.05E-08 | 478,149 | 30.678 |
| rs116099394 | T | G | 0.146 | -0.058 | 0.008 | 8.70E-15 | 478,149 | 60.169 |
| rs2704368 | G | A | 0.837 | -0.064 | 0.007 | 6.63E-19 | 478,149 | 78.871 |
| rs7580802 | C | G | 0.104 | -0.060 | 0.009 | 5.31E-12 | 478,149 | 47.571 |
| rs4362519 | C | G | 0.362 | -0.032 | 0.006 | 3.92E-09 | 478,149 | 34.665 |
| rs16828714 | T | G | 0.358 | -0.031 | 0.006 | 2.61E-08 | 478,149 | 30.981 |
| rs115886992 | C | T | 0.034 | -0.082 | 0.015 | 1.97E-08 | 478,149 | 31.526 |
| rs78058190 | A | G | 0.082 | 0.056 | 0.010 | 5.94E-09 | 478,149 | 33.855 |
| rs1515114 | G | A | 0.428 | 0.035 | 0.005 | 3.89E-11 | 478,149 | 43.671 |
| rs2574698 | A | G | 0.578 | 0.034 | 0.005 | 2.06E-10 | 478,149 | 40.413 |
| rs1687320 | G | C | 0.227 | 0.038 | 0.006 | 2.60E-09 | 478,149 | 35.466 |
| rs293927 | G | A | 0.809 | -0.038 | 0.007 | 2.02E-08 | 478,149 | 31.480 |
| rs2643826 | T | C | 0.403 | 0.047 | 0.005 | 2.43E-18 | 478,149 | 76.303 |
| rs2271487 | G | A | 0.493 | 0.031 | 0.005 | 7.22E-09 | 478,149 | 33.476 |
| rs3821843 | A | G | 0.659 | 0.035 | 0.006 | 3.15E-10 | 478,149 | 39.578 |
| rs9836592 | T | C | 0.651 | 0.037 | 0.006 | 2.76E-11 | 478,149 | 44.343 |
| rs73226528 | C | T | 0.282 | 0.033 | 0.006 | 1.68E-08 | 478,149 | 31.830 |
| rs9851392 | A | T | 0.551 | 0.037 | 0.005 | 2.96E-12 | 478,149 | 48.712 |
| rs9844972 | C | G | 0.054 | 0.070 | 0.012 | 1.69E-09 | 478,149 | 36.300 |
| rs56279242 | T | C | 0.443 | 0.030 | 0.005 | 2.22E-08 | 478,149 | 31.288 |
| rs6808266 | G | A | 0.799 | 0.048 | 0.007 | 5.02E-13 | 478,149 | 52.198 |
| rs9865831 | G | T | 0.079 | -0.058 | 0.010 | 3.26E-09 | 478,149 | 35.022 |
| rs10804833 | A | G | 0.497 | -0.031 | 0.005 | 8.06E-09 | 478,149 | 33.261 |
| rs55924432 | T | C | 0.478 | 0.032 | 0.005 | 2.15E-09 | 478,149 | 35.836 |
| rs11721984 | T | C | 0.364 | -0.031 | 0.006 | 1.45E-08 | 478,149 | 32.116 |
| rs55893990 | G | A | 0.318 | 0.034 | 0.006 | 2.54E-09 | 478,149 | 35.512 |
| rs12509595 | C | T | 0.313 | 0.088 | 0.006 | 3.08E-53 | 478,149 | 235.899 |
| rs17010957 | C | T | 0.152 | 0.051 | 0.007 | 3.95E-12 | 478,149 | 48.149 |
| rs13112725 | C | G | 0.828 | 0.054 | 0.007 | 1.55E-14 | 478,149 | 59.034 |
| rs2001255 | T | C | 0.495 | -0.039 | 0.005 | 3.19E-13 | 478,149 | 53.090 |
| rs1836039 | G | A | 0.149 | -0.046 | 0.007 | 8.73E-10 | 478,149 | 37.590 |
| rs76386877 | C | T | 0.469 | 0.029 | 0.005 | 3.36E-08 | 478,149 | 30.488 |
| rs4690777 | C | T | 0.644 | -0.035 | 0.006 | 3.28E-10 | 478,149 | 39.500 |
| rs1021956 | T | G | 0.857 | -0.044 | 0.008 | 8.62E-09 | 478,149 | 33.129 |
| rs4691670 | T | C | 0.400 | -0.036 | 0.005 | 4.33E-11 | 478,149 | 43.461 |
| rs10029150 | G | C | 0.774 | 0.072 | 0.006 | 3.65E-30 | 478,149 | 130.228 |
| rs60776549 | G | A | 0.275 | 0.037 | 0.006 | 4.89E-10 | 478,149 | 38.719 |
| rs12656497 | C | T | 0.585 | 0.056 | 0.005 | 1.58E-25 | 478,149 | 109.054 |
| rs188155432 | T | C | 0.024 | 0.138 | 0.017 | 1.59E-15 | 478,149 | 63.516 |
| rs3857275 | C | T | 0.256 | -0.034 | 0.006 | 2.27E-08 | 478,149 | 31.248 |
| rs10050467 | T | C | 0.408 | -0.031 | 0.005 | 6.76E-09 | 478,149 | 33.602 |
| rs1819074 | A | G | 0.666 | -0.034 | 0.006 | 1.07E-09 | 478,149 | 37.188 |
| rs2549805 | A | T | 0.808 | 0.055 | 0.007 | 4.23E-16 | 478,149 | 66.125 |
| rs10077410 | A | G | 0.226 | 0.040 | 0.006 | 1.38E-10 | 478,149 | 41.191 |
| rs6860901 | T | C | 0.297 | 0.056 | 0.006 | 1.65E-22 | 478,149 | 95.287 |
| rs982085 | G | C | 0.549 | 0.032 | 0.005 | 1.85E-09 | 478,149 | 36.131 |
| rs4554204 | A | G | 0.385 | 0.033 | 0.005 | 9.22E-10 | 478,149 | 37.483 |
| rs10042748 | T | G | 0.331 | -0.034 | 0.006 | 2.40E-09 | 478,149 | 35.620 |
| rs4371736 | C | G | 0.382 | -0.047 | 0.005 | 6.05E-18 | 478,149 | 74.503 |
| rs1345721 | C | G | 0.185 | -0.043 | 0.007 | 2.81E-10 | 478,149 | 39.801 |
| rs4286697 | G | A | 0.644 | 0.031 | 0.006 | 1.47E-08 | 478,149 | 32.095 |
| rs2569882 | C | T | 0.376 | -0.031 | 0.005 | 9.07E-09 | 478,149 | 33.030 |
| rs198833 | A | G | 0.889 | -0.064 | 0.008 | 2.20E-14 | 478,149 | 58.347 |
| rs3131004 | G | A | 0.450 | 0.045 | 0.005 | 3.66E-17 | 478,149 | 70.953 |
| rs3993747 | G | A | 0.320 | 0.036 | 0.006 | 2.27E-10 | 478,149 | 40.218 |
| rs115493740 | A | G | 0.105 | 0.062 | 0.009 | 9.41E-13 | 478,149 | 50.963 |
| rs7742789 | T | C | 0.294 | 0.050 | 0.006 | 3.43E-18 | 478,149 | 75.627 |
| rs1358980 | T | C | 0.463 | 0.035 | 0.005 | 2.21E-11 | 478,149 | 44.776 |
| rs4715224 | A | T | 0.398 | 0.039 | 0.005 | 3.56E-13 | 478,149 | 52.873 |
| rs1415859 | G | A | 0.410 | 0.045 | 0.005 | 9.09E-17 | 478,149 | 69.156 |
| rs6918791 | G | C | 0.728 | 0.044 | 0.006 | 2.11E-13 | 478,149 | 53.904 |
| rs62426324 | T | C | 0.500 | 0.059 | 0.005 | 8.65E-29 | 478,149 | 123.948 |
| rs198645 | T | A | 0.332 | -0.042 | 0.006 | 8.98E-14 | 478,149 | 55.578 |
| rs17080102 | C | G | 0.076 | -0.099 | 0.010 | 7.08E-23 | 478,149 | 96.958 |
| rs73030239 | G | A | 0.056 | -0.067 | 0.012 | 8.15E-09 | 478,149 | 33.240 |
| rs11973114 | A | G | 0.242 | -0.038 | 0.006 | 8.91E-10 | 478,149 | 37.550 |
| rs4724958 | G | A | 0.443 | 0.033 | 0.005 | 4.47E-10 | 478,149 | 38.897 |
| rs7793945 | A | G | 0.150 | 0.052 | 0.007 | 2.64E-12 | 478,149 | 48.939 |
| rs3735533 | C | T | 0.904 | 0.127 | 0.009 | 4.65E-44 | 478,149 | 193.827 |
| rs11563582 | A | G | 0.146 | 0.072 | 0.008 | 8.16E-22 | 478,149 | 92.119 |
| rs12540319 | C | T | 0.036 | 0.080 | 0.014 | 1.72E-08 | 478,149 | 31.793 |
| rs17492269 | A | G | 0.231 | -0.039 | 0.006 | 6.85E-10 | 478,149 | 38.062 |
| rs67763033 | G | A | 0.195 | 0.041 | 0.007 | 6.29E-10 | 478,149 | 38.228 |
| rs17688839 | T | A | 0.204 | -0.036 | 0.007 | 3.04E-08 | 478,149 | 30.683 |
| rs472660 | A | G | 0.117 | -0.046 | 0.008 | 1.91E-08 | 478,149 | 31.579 |
| rs17477177 | C | T | 0.298 | 0.052 | 0.006 | 2.60E-19 | 478,149 | 80.721 |
| rs10261050 | T | C | 0.468 | 0.029 | 0.005 | 3.51E-08 | 478,149 | 30.404 |
| rs193686 | T | C | 0.719 | -0.040 | 0.006 | 1.79E-11 | 478,149 | 45.185 |
| rs75511781 | G | A | 0.079 | 0.067 | 0.010 | 8.89E-12 | 478,149 | 46.559 |
| rs57274670 | C | G | 0.265 | -0.039 | 0.006 | 1.40E-10 | 478,149 | 41.161 |
| rs3918226 | T | C | 0.070 | 0.160 | 0.010 | 6.44E-55 | 478,149 | 243.604 |
| rs10253736 | T | C | 0.231 | 0.045 | 0.006 | 6.33E-13 | 478,149 | 51.741 |
| rs62494194 | A | G | 0.450 | 0.031 | 0.005 | 3.69E-09 | 478,149 | 34.779 |
| rs2442466 | G | C | 0.505 | 0.030 | 0.005 | 1.76E-08 | 478,149 | 31.746 |
| rs7831557 | A | G | 0.467 | -0.044 | 0.005 | 1.29E-16 | 478,149 | 68.460 |
| rs10086575 | A | G | 0.296 | -0.039 | 0.006 | 1.91E-11 | 478,149 | 45.058 |
| rs17310011 | G | C | 0.545 | 0.033 | 0.005 | 3.36E-10 | 478,149 | 39.453 |
| rs116848980 | A | G | 0.056 | -0.066 | 0.012 | 1.94E-08 | 478,149 | 31.558 |
| rs2920921 | G | A | 0.718 | 0.033 | 0.006 | 1.47E-08 | 478,149 | 32.086 |
| rs2515236 | G | A | 0.391 | 0.037 | 0.005 | 8.54E-12 | 478,149 | 46.638 |
| rs35783704 | A | G | 0.155 | -0.066 | 0.007 | 4.34E-19 | 478,149 | 79.706 |
| rs3802228 | G | A | 0.569 | -0.035 | 0.005 | 3.72E-11 | 478,149 | 43.754 |
| rs628919 | A | C | 0.540 | -0.032 | 0.005 | 1.60E-09 | 478,149 | 36.404 |
| rs1412834 | C | T | 0.434 | 0.044 | 0.005 | 7.37E-17 | 478,149 | 69.572 |
| rs7874497 | G | A | 0.628 | 0.040 | 0.006 | 2.35E-13 | 478,149 | 53.686 |
| rs67227401 | T | C | 0.100 | -0.056 | 0.009 | 3.33E-10 | 478,149 | 39.469 |
| rs10217559 | T | C | 0.693 | 0.037 | 0.006 | 1.54E-10 | 478,149 | 40.980 |
| rs12375937 | C | G | 0.089 | 0.058 | 0.009 | 5.32E-10 | 478,149 | 38.555 |
| rs9696459 | T | C | 0.319 | -0.032 | 0.006 | 2.41E-08 | 478,149 | 31.135 |
| rs72779268 | A | C | 0.064 | -0.115 | 0.011 | 5.21E-26 | 478,149 | 111.253 |
| rs10739995 | G | A | 0.121 | 0.050 | 0.008 | 7.14E-10 | 478,149 | 37.983 |
| rs1888693 | A | G | 0.386 | 0.038 | 0.005 | 4.18E-12 | 478,149 | 48.038 |
| rs188460025 | T | C | 0.070 | -0.074 | 0.010 | 1.13E-12 | 478,149 | 50.610 |
| rs72831343 | G | T | 0.102 | -0.065 | 0.009 | 9.17E-14 | 478,149 | 55.537 |
| rs1848797 | G | A | 0.358 | -0.032 | 0.006 | 4.21E-09 | 478,149 | 34.525 |
| rs3740051 | G | A | 0.130 | 0.049 | 0.008 | 4.71E-10 | 478,149 | 38.793 |
| rs1248670 | G | A | 0.613 | 0.030 | 0.005 | 2.60E-08 | 478,149 | 30.984 |
| rs2274224 | C | G | 0.345 | -0.044 | 0.006 | 3.04E-15 | 478,149 | 62.240 |
| rs12411886 | A | C | 0.085 | -0.100 | 0.010 | 1.12E-25 | 478,149 | 109.738 |
| rs7919263 | T | C | 0.292 | 0.032 | 0.006 | 4.71E-08 | 478,149 | 29.832 |
| rs1265002 | T | A | 0.953 | -0.092 | 0.012 | 1.71E-13 | 478,149 | 54.308 |
| rs7920508 | G | A | 0.311 | -0.039 | 0.006 | 1.58E-11 | 478,149 | 45.437 |
| rs2782980 | C | T | 0.654 | 0.058 | 0.006 | 2.73E-25 | 478,149 | 107.968 |
| rs3781433 | A | G | 0.579 | -0.030 | 0.005 | 2.88E-08 | 478,149 | 30.790 |
| rs612652 | C | T | 0.573 | 0.058 | 0.005 | 2.34E-27 | 478,149 | 117.409 |
| rs55640925 | A | C | 0.175 | -0.051 | 0.007 | 3.51E-13 | 478,149 | 52.900 |
| rs72851658 | A | T | 0.197 | 0.055 | 0.007 | 1.65E-16 | 478,149 | 67.984 |
| rs11030017 | T | C | 0.871 | -0.061 | 0.008 | 8.32E-15 | 478,149 | 60.258 |
| rs34906813 | G | A | 0.265 | 0.042 | 0.006 | 4.08E-12 | 478,149 | 48.088 |
| rs10835544 | C | A | 0.316 | 0.032 | 0.006 | 1.67E-08 | 478,149 | 31.844 |
| rs2868996 | T | A | 0.238 | 0.042 | 0.006 | 2.18E-11 | 478,149 | 44.805 |
| rs7106223 | A | G | 0.191 | -0.039 | 0.007 | 6.07E-09 | 478,149 | 33.812 |
| rs35094231 | C | G | 0.079 | -0.061 | 0.010 | 5.41E-10 | 478,149 | 38.525 |
| rs751984 | C | T | 0.171 | -0.066 | 0.007 | 5.42E-21 | 478,149 | 88.374 |
| rs7127546 | A | G | 0.468 | -0.030 | 0.005 | 1.24E-08 | 478,149 | 32.423 |
| rs4246958 | C | T | 0.499 | -0.029 | 0.005 | 3.43E-08 | 478,149 | 30.449 |
| rs604723 | C | T | 0.740 | 0.066 | 0.006 | 1.98E-27 | 478,149 | 117.733 |
| rs10047462 | T | G | 0.838 | -0.047 | 0.007 | 5.22E-11 | 478,149 | 43.094 |
| rs665759 | A | G | 0.250 | 0.034 | 0.006 | 2.47E-08 | 478,149 | 31.084 |
| rs11600607 | G | A | 0.154 | 0.042 | 0.007 | 1.36E-08 | 478,149 | 32.242 |
| rs12828438 | G | A | 0.535 | -0.042 | 0.005 | 2.85E-15 | 478,149 | 62.368 |
| rs10466855 | A | G | 0.269 | 0.034 | 0.006 | 1.15E-08 | 478,149 | 32.571 |
| rs114850651 | A | G | 0.039 | -0.085 | 0.014 | 6.14E-10 | 478,149 | 38.277 |
| rs73073676 | T | A | 0.220 | -0.043 | 0.006 | 1.38E-11 | 478,149 | 45.697 |
| rs7967954 | A | G | 0.590 | -0.043 | 0.005 | 1.63E-15 | 478,149 | 63.469 |
| rs78204022 | T | C | 0.053 | 0.070 | 0.012 | 2.91E-09 | 478,149 | 35.241 |
| rs2010844 | G | T | 0.604 | 0.030 | 0.005 | 3.29E-08 | 478,149 | 30.531 |
| rs2293059 | A | G | 0.075 | -0.060 | 0.010 | 2.13E-09 | 478,149 | 35.855 |
| rs7139122 | A | G | 0.020 | 0.198 | 0.019 | 3.75E-26 | 478,149 | 111.902 |
| rs12319419 | C | G | 0.408 | -0.058 | 0.005 | 2.47E-27 | 478,149 | 117.299 |
| rs11177594 | T | C | 0.199 | -0.036 | 0.007 | 4.34E-08 | 478,149 | 29.993 |
| rs117703053 | A | G | 0.093 | 0.050 | 0.009 | 3.65E-08 | 478,149 | 30.329 |
| rs11105337 | T | A | 0.077 | -0.095 | 0.010 | 1.66E-21 | 478,149 | 90.715 |
| rs3184504 | C | T | 0.593 | -0.066 | 0.005 | 9.77E-35 | 478,149 | 151.138 |
| rs35429 | G | A | 0.365 | -0.046 | 0.006 | 7.01E-17 | 478,149 | 69.671 |
| rs6490021 | C | A | 0.696 | 0.047 | 0.006 | 5.77E-16 | 478,149 | 65.514 |
| rs507446 | T | C | 0.579 | 0.040 | 0.005 | 6.38E-14 | 478,149 | 56.251 |
| rs9508490 | T | C | 0.642 | -0.036 | 0.006 | 5.49E-11 | 478,149 | 42.996 |
| rs1460451 | C | T | 0.467 | -0.031 | 0.005 | 6.36E-09 | 478,149 | 33.721 |
| rs9590507 | C | T | 0.212 | 0.040 | 0.006 | 7.09E-10 | 478,149 | 37.997 |
| rs117909153 | T | C | 0.081 | 0.054 | 0.010 | 3.08E-08 | 478,149 | 30.655 |
| rs1136165 | T | G | 0.680 | 0.038 | 0.006 | 2.61E-11 | 478,149 | 44.452 |
| rs79622589 | T | C | 0.221 | -0.038 | 0.006 | 3.95E-09 | 478,149 | 34.649 |
| rs2925345 | C | T | 0.649 | -0.042 | 0.006 | 2.61E-14 | 478,149 | 58.011 |
| rs12911761 | T | C | 0.398 | 0.033 | 0.005 | 1.33E-09 | 478,149 | 36.771 |
| rs1984442 | C | T | 0.644 | 0.038 | 0.006 | 8.70E-12 | 478,149 | 46.601 |
| rs117539635 | G | A | 0.017 | -0.115 | 0.021 | 4.09E-08 | 478,149 | 30.105 |
| rs2759315 | A | C | 0.400 | 0.043 | 0.005 | 1.07E-15 | 478,149 | 64.300 |
| rs17807723 | A | G | 0.190 | -0.045 | 0.007 | 2.23E-11 | 478,149 | 44.761 |
| rs35346340 | C | G | 0.271 | 0.070 | 0.006 | 1.13E-31 | 478,149 | 137.123 |
| rs35198836 | T | C | 0.321 | 0.036 | 0.006 | 1.34E-10 | 478,149 | 41.243 |
| rs117645982 | G | C | 0.023 | 0.112 | 0.018 | 3.18E-10 | 478,149 | 39.563 |
| rs77924615 | A | G | 0.223 | -0.036 | 0.006 | 1.94E-08 | 478,149 | 31.551 |
| rs9937354 | A | G | 0.428 | 0.062 | 0.005 | 6.99E-31 | 478,149 | 133.511 |
| rs45474499 | T | C | 0.063 | 0.070 | 0.011 | 9.67E-11 | 478,149 | 41.887 |
| rs10871309 | T | C | 0.386 | 0.030 | 0.005 | 4.83E-08 | 478,149 | 29.785 |
| rs2966083 | T | G | 0.587 | -0.030 | 0.005 | 2.36E-08 | 478,149 | 31.176 |
| rs8045875 | C | T | 0.594 | 0.040 | 0.005 | 1.75E-13 | 478,149 | 54.273 |
| rs11645355 | A | G | 0.293 | 0.038 | 0.006 | 4.96E-11 | 478,149 | 43.194 |
| rs4796520 | G | T | 0.550 | 0.035 | 0.005 | 4.72E-11 | 478,149 | 43.290 |
| rs5418 | A | G | 0.602 | 0.046 | 0.005 | 8.58E-18 | 478,149 | 73.816 |
| rs7223364 | T | G | 0.172 | 0.038 | 0.007 | 4.60E-08 | 478,149 | 29.879 |
| rs4792967 | G | A | 0.581 | -0.032 | 0.005 | 3.39E-09 | 478,149 | 34.948 |
| rs117953218 | C | T | 0.170 | 0.061 | 0.007 | 4.33E-18 | 478,149 | 75.163 |
| rs11650158 | A | G | 0.207 | -0.038 | 0.007 | 6.31E-09 | 478,149 | 33.735 |
| rs9897429 | A | G | 0.517 | 0.035 | 0.005 | 6.33E-11 | 478,149 | 42.715 |
| rs2257205 | T | C | 0.142 | -0.043 | 0.008 | 1.49E-08 | 478,149 | 32.064 |
| rs35619711 | G | C | 0.721 | 0.051 | 0.006 | 3.42E-18 | 478,149 | 75.628 |
| rs75646162 | A | G | 0.079 | -0.062 | 0.010 | 4.34E-10 | 478,149 | 38.954 |
| rs1436138 | G | A | 0.356 | -0.035 | 0.006 | 3.73E-10 | 478,149 | 39.250 |
| rs303753 | A | G | 0.351 | 0.035 | 0.006 | 1.99E-10 | 478,149 | 40.481 |
| rs11083117 | C | T | 0.115 | 0.046 | 0.008 | 2.34E-08 | 478,149 | 31.188 |
| rs4599004 | T | C | 0.286 | -0.038 | 0.006 | 5.98E-11 | 478,149 | 42.827 |
| rs146847723 | C | T | 0.141 | 0.045 | 0.008 | 3.37E-09 | 478,149 | 34.957 |
| rs2738449 | T | C | 0.469 | 0.029 | 0.005 | 3.49E-08 | 478,149 | 30.414 |
| rs143466522 | A | G | 0.015 | 0.174 | 0.022 | 2.27E-15 | 478,149 | 62.817 |
| rs167479 | G | T | 0.576 | 0.074 | 0.005 | 4.54E-43 | 478,149 | 189.290 |
| rs11667341 | T | C | 0.033 | -0.093 | 0.015 | 4.54E-10 | 478,149 | 38.863 |
| rs2278997 | A | G | 0.218 | 0.043 | 0.006 | 1.19E-11 | 478,149 | 45.986 |
| rs2005873 | T | C | 0.552 | -0.030 | 0.005 | 1.24E-08 | 478,149 | 32.417 |
| rs376510895 | A | G | 0.023 | -0.103 | 0.018 | 1.10E-08 | 478,149 | 32.649 |
| rs6108165 | A | G | 0.209 | -0.037 | 0.007 | 1.73E-08 | 478,149 | 31.781 |
| rs6040076 | C | G | 0.433 | -0.030 | 0.005 | 2.13E-08 | 478,149 | 31.368 |
| rs62185661 | A | G | 0.242 | 0.055 | 0.006 | 4.51E-19 | 478,149 | 79.634 |
| rs12481717 | A | G | 0.194 | 0.043 | 0.007 | 1.08E-10 | 478,149 | 41.669 |
| rs6017285 | G | C | 0.125 | 0.051 | 0.008 | 1.79E-10 | 478,149 | 40.679 |
| rs11906755 | G | A | 0.164 | 0.118 | 0.007 | 1.77E-61 | 478,149 | 273.700 |
| rs9679855 | G | A | 0.033 | 0.111 | 0.015 | 2.57E-14 | 478,149 | 58.039 |
| rs180957609 | C | A | 0.034 | -0.090 | 0.015 | 1.07E-09 | 478,149 | 37.200 |
| rs2427541 | G | C | 0.501 | -0.038 | 0.005 | 6.56E-13 | 478,149 | 51.672 |
| rs2823140 | A | G | 0.384 | 0.035 | 0.005 | 1.81E-10 | 478,149 | 40.659 |
| rs541476 | T | C | 0.231 | 0.038 | 0.006 | 2.88E-09 | 478,149 | 35.263 |
| rs6518309 | A | C | 0.687 | 0.037 | 0.006 | 9.22E-11 | 478,149 | 41.980 |
| rs5762879 | T | C | 0.212 | 0.053 | 0.006 | 3.10E-16 | 478,149 | 66.741 |
| rs134557 | G | A | 0.779 | 0.035 | 0.006 | 3.00E-08 | 478,149 | 30.707 |

**Table S6. 28 SNPs associated with pregnancy hypertension.**

| SNP | Effect  allele | Other allele | Effect allele  frequency | Beta | Se | Pval | Sample  size | F |
| --- | --- | --- | --- | --- | --- | --- | --- | --- |
| rs13306561 | G | A | 0.145 | -0.136 | 0.015 | 2.37E-19 | 282,064 | 80.901 |
| rs7589428 | G | A | 0.490 | -0.058 | 0.010 | 2.07E-08 | 282,064 | 31.426 |
| rs2011946 | A | C | 0.646 | 0.066 | 0.011 | 1.93E-09 | 282,064 | 36.041 |
| rs11917304 | C | T | 0.136 | -0.091 | 0.015 | 3.73E-09 | 282,064 | 34.758 |
| rs9855086 | T | A | 0.462 | -0.080 | 0.010 | 1.66E-14 | 282,064 | 58.904 |
| rs12509595 | C | T | 0.313 | 0.127 | 0.011 | 2.77E-30 | 282,064 | 130.774 |
| rs10001721 | T | C | 0.317 | 0.064 | 0.011 | 1.03E-08 | 282,064 | 32.778 |
| rs12656497 | C | T | 0.586 | 0.095 | 0.011 | 3.61E-19 | 282,064 | 80.075 |
| rs4413520 | C | A | 0.926 | -0.107 | 0.019 | 4.12E-08 | 282,064 | 30.094 |
| rs9271710 | A | G | 0.675 | 0.069 | 0.011 | 7.31E-10 | 282,064 | 37.935 |
| rs1053538 | G | C | 0.411 | 0.059 | 0.011 | 1.95E-08 | 282,064 | 31.543 |
| rs1554446 | C | T | 0.283 | -0.064 | 0.012 | 3.54E-08 | 282,064 | 30.388 |
| rs4721153 | G | C | 0.505 | -0.057 | 0.010 | 4.33E-08 | 282,064 | 29.996 |
| rs73672446 | T | C | 0.093 | 0.108 | 0.017 | 4.80E-10 | 282,064 | 38.758 |
| rs12258967 | G | C | 0.221 | -0.071 | 0.013 | 2.26E-08 | 282,064 | 31.261 |
| rs10882398 | A | T | 0.593 | 0.078 | 0.011 | 2.58E-13 | 282,064 | 53.505 |
| rs2782981 | C | T | 0.654 | 0.069 | 0.011 | 3.45E-10 | 282,064 | 39.403 |
| rs604723 | C | T | 0.741 | 0.080 | 0.012 | 2.73E-11 | 282,064 | 44.364 |
| rs7139122 | A | G | 0.021 | 0.273 | 0.033 | 2.89E-16 | 282,064 | 66.879 |
| rs12230074 | G | A | 0.075 | -0.122 | 0.020 | 1.92E-09 | 282,064 | 36.049 |
| rs7137828 | T | C | 0.587 | -0.079 | 0.010 | 6.24E-14 | 282,064 | 56.293 |
| rs12591078 | C | T | 0.711 | -0.066 | 0.011 | 5.73E-09 | 282,064 | 33.923 |
| rs4932376 | G | C | 0.415 | -0.071 | 0.011 | 2.65E-11 | 282,064 | 44.420 |
| rs11642015 | T | C | 0.416 | 0.070 | 0.010 | 2.76E-11 | 282,064 | 44.338 |
| rs167479 | G | T | 0.576 | 0.100 | 0.011 | 2.16E-21 | 282,064 | 90.198 |
| rs35591645 | T | C | 0.045 | 0.134 | 0.024 | 3.50E-08 | 282,064 | 30.411 |
| rs2208589 | G | A | 0.890 | 0.106 | 0.017 | 6.96E-10 | 282,064 | 38.031 |
| rs6026739 | T | A | 0.170 | 0.127 | 0.013 | 3.69E-21 | 282,064 | 89.134 |

**Table S7. 19 SNPs associated with hypertensive heart disease.**

| SNP | Effect  allele | Other allele | Effect allele  frequency | Beta | Se | Pval | Sample  size | F |
| --- | --- | --- | --- | --- | --- | --- | --- | --- |
| rs17035646 | A | G | 0.393 | 0.125 | 0.014 | 2.49E-18 | 357,362 | 76.260 |
| rs3790604 | A | C | 0.169 | 0.138 | 0.019 | 8.35E-14 | 357,362 | 55.721 |
| rs35021474 | G | C | 0.553 | -0.111 | 0.014 | 4.23E-15 | 357,362 | 61.589 |
| rs10185395 | T | G | 0.242 | 0.091 | 0.016 | 2.89E-08 | 357,362 | 30.780 |
| rs16998073 | T | A | 0.310 | 0.097 | 0.015 | 1.63E-10 | 357,362 | 40.863 |
| rs13118687 | A | G | 0.487 | -0.094 | 0.014 | 2.72E-11 | 357,362 | 44.372 |
| rs73124033 | T | G | 0.142 | 0.113 | 0.020 | 1.09E-08 | 357,362 | 32.668 |
| rs1092414 | C | T | 0.581 | -0.093 | 0.014 | 6.43E-11 | 357,362 | 42.687 |
| rs1268792 | A | G | 0.434 | -0.088 | 0.014 | 7.84E-10 | 357,362 | 37.798 |
| rs73073444 | T | C | 0.036 | 0.205 | 0.037 | 2.40E-08 | 357,362 | 31.144 |
| rs3735533 | C | T | 0.903 | 0.167 | 0.025 | 8.76E-12 | 357,362 | 46.589 |
| rs17398575 | A | G | 0.316 | 0.088 | 0.015 | 5.54E-09 | 357,362 | 33.991 |
| rs12705986 | G | A | 0.430 | 0.080 | 0.014 | 1.89E-08 | 357,362 | 31.605 |
| rs3918226 | T | C | 0.068 | 0.201 | 0.027 | 1.55E-13 | 357,362 | 54.511 |
| rs7874497 | G | A | 0.627 | 0.090 | 0.015 | 8.37E-10 | 357,362 | 37.672 |
| rs661348 | C | T | 0.470 | 0.106 | 0.014 | 4.19E-14 | 357,362 | 57.075 |
| rs6580970 | T | C | 0.394 | -0.086 | 0.014 | 3.39E-09 | 357,362 | 34.946 |
| rs56310356 | G | C | 0.401 | 0.083 | 0.014 | 8.22E-09 | 357,362 | 33.223 |
| rs9937354 | A | G | 0.426 | 0.082 | 0.014 | 7.28E-09 | 357,362 | 33.459 |
